# Triple lysine and nucleosome-binding motifs of the viral IE19 protein are required for human cytomegalovirus S-phase infections

**DOI:** 10.1128/mbio.00162-24

**Published:** 2024-05-02

**Authors:** Minor R. Maliano, Kristen D. Yetming, Robert F. Kalejta

**Affiliations:** 1 Institute for Molecular Virology, University of Wisconsin–Madison, Madison, Wisconsin, USA; 2 McArdle Laboratory for Cancer Research, University of Wisconsin–Madison, Madison, Wisconsin, USA; 3 Molecular Biology, Charles River Laboratories, Wayne, Pennsylvania, USA; Princeton University, Princeton, New Jersey, USA

**Keywords:** mitosis, herpesviruses, chromatin, cancer, genome maintenance

## Abstract

Herpesvirus genomes are maintained as extrachromosomal plasmids within the nuclei of infected cells. Some herpesviruses persist within dividing cells, putting the viral genome at risk of being lost to the cytoplasm during mitosis because karyokinesis (nuclear division) requires nuclear envelope breakdown. Oncogenic herpesviruses (and papillomaviruses) avoid genome loss during mitosis by tethering their genomes to cellular chromosomes, thereby ensuring viral genome uptake into newly formed nuclei. These viruses use viral proteins with DNA- and chromatin-binding capabilities to physically link viral and cellular genomes together in a process called tethering. The known viral tethering proteins of human papillomavirus (E2), Epstein-Barr virus (EBNA1), and Kaposi’s sarcoma-associated herpesvirus (LANA) each contain two independent domains required for genome tethering, one that binds sequence specifically to the viral genome and another that binds to cellular chromatin. This latter domain is called a chromatin tethering domain (CTD). The human cytomegalovirus UL123 gene encodes a CTD that is required for the virus to productively infect dividing fibroblast cells within the S phase of the cell cycle, presumably by tethering the viral genome to cellular chromosomes during mitosis. The CTD-containing UL123 gene product that supports S-phase infections is the IE19 protein. Here, we define two motifs in IE19 required for S-phase infections: an N-terminal triple lysine motif and a C-terminal nucleosome-binding motif within the CTD.

**IMPORTANCE:**

The IE19 protein encoded by human cytomegalovirus (HCMV) is required for S-phase infection of dividing cells, likely because it tethers the viral genome to cellular chromosomes, thereby allowing them to survive mitosis. The mechanism through which IE19 tethers viral genomes to cellular chromosomes is not understood. For human papillomavirus, Epstein-Barr virus, and Kaposi’s sarcoma-associated herpesvirus, viral genome tethering is required for persistence (latency) and pathogenesis (oncogenesis). Like these viruses, HCMV also achieves latency, and it modulates the properties of glioblastoma multiforme tumors. Therefore, defining the mechanism through which IE19 tethers viral genomes to cellular chromosomes may help us understand, and ultimately combat or control, HCMV latency and oncomodulation.

## INTRODUCTION

Viral infections are acute (resolved in days or weeks, e.g., influenza), persistent (resolved in months, years, or decades, e.g., papillomaviruses), or latent (lifelong, e.g., herpesviruses). Persistent or latent dsDNA viruses maintain their genomes in the nucleus without making infectious progeny virions that would trigger adaptive immune responses (1–5). Mitosis represents a significant problem for persistent or latent dsDNA viruses that maintain their genomes as extrachromosomal elements in the nuclei of dividing cells. The nuclear envelope dissolves during mitosis, allowing extrachromosomal DNA to diffuse throughout the cell. Extra-chromosomal viral genomes lack centromeres. Those not serendipitously captured by reforming nuclear envelopes would be confined to the cytoplasm where they are incapable of sustaining infection or re-entering the nucleus, and where they would elicit antiviral innate immune responses. Human papillomaviruses (HPVs) and gamma-herpesviruses [Epstein-Barr virus (EBV) and Kaposi’s sarcoma-associated herpesvirus (KSHV)] maintain their genomes as extrachromosomal plasmids in the nuclei of dividing cells but avoid viral genome loss during mitosis because they non-covalently attach their genomes to cellular chromosomes using viral tethering proteins (3–7). Viral tethering proteins simultaneously bind viral genomes and cellular chromosomes and physically link the two so that the viral genomes are sure to be incorporated into newly forming nuclei in the nascent daughter cells. HPV, EBV, and KSHV each encode tethering proteins (HPV E2, EBV EBNA1, and KSHV LANA) that dimerize or oligomerize (3, 4, 8–13). Other common features (3–5) of viral tethering proteins include a C-terminal sequence-specific DNA-binding domain that associates with the viral genome and an N-terminal chromatin tethering domain (CTD) that associates with cellular chromosomes (3–7). Colocalization with mitotic chromosomes, a defining feature of tethering proteins, was first described for EBNA1 (14). The immediate early one (IE1) protein, encoded by the UL123 gene of HCMV, was the second viral protein found to associate with mitotic chromosomes (15).

IE1 is a 72 kDa, 491 amino acid protein that represents the major activator of HCMV productive infection (16). Unlike the other known viral tethers, its CTD is found at the carboxy terminus of the protein (17–19). The IE1 CTD is dispensable for productive infection in unsynchronized or nearly confluent fibroblasts in both laboratory (18, 19) and clinical strains of HCMV (20) and binds to the acidic pocket between nucleosomal histones H2A and H2B in a manner similar to KSHV LANA (20, 21). The putative function of the CTD is to tether HCMV genomes to cellular chromosomes (15, 16). Indeed, deletion of the UL123 gene or just the CTD reduces the number of viral genomes found adjacent to cellular chromosomes in metaphase spreads (22). The role of such tethering is presumably to allow the viral genome to survive mitosis in a dividing cell (15, 16).

**Fig 1:**
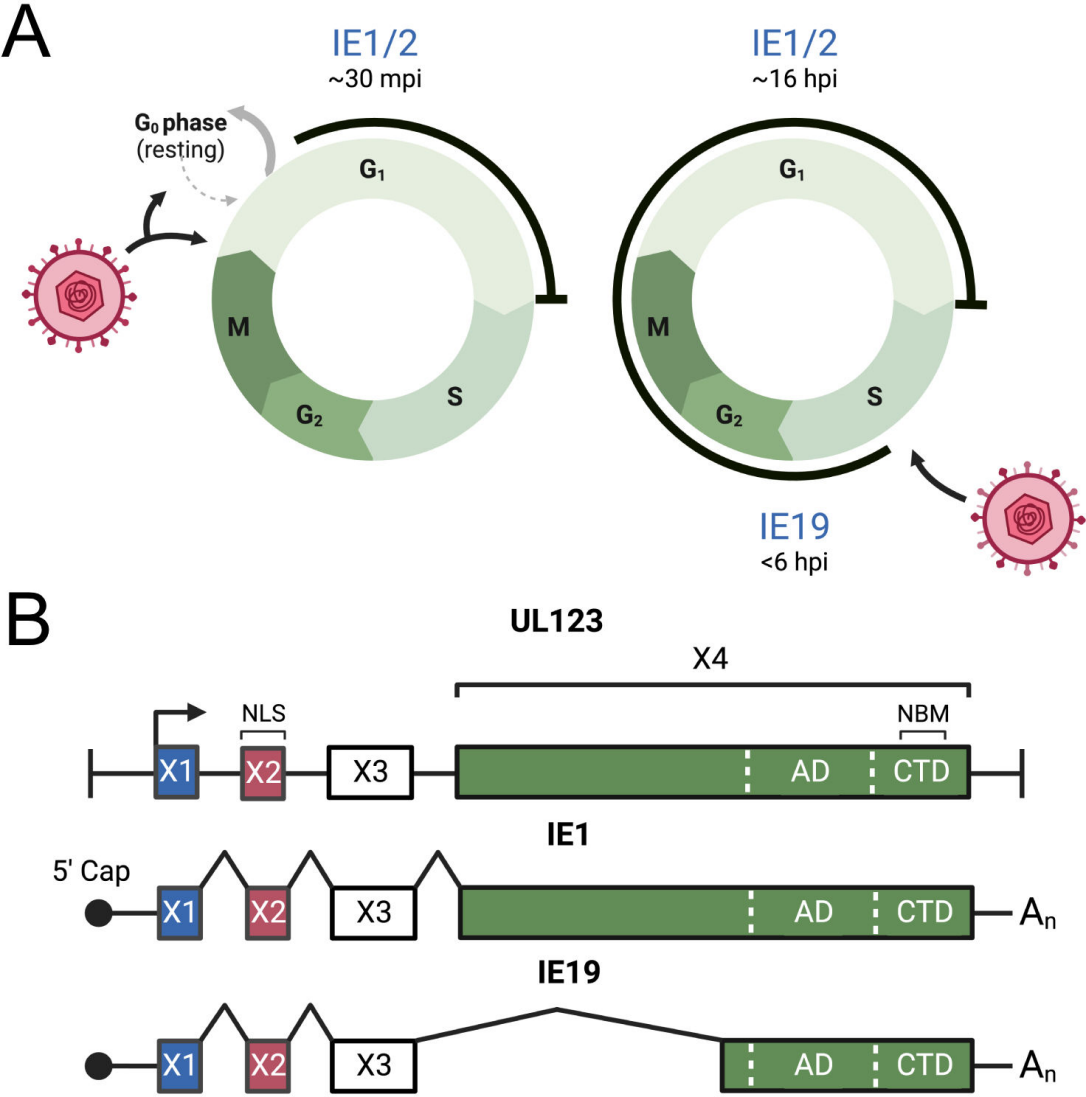
The HCMV IE19 protein is required for S-phase infections. (**A**) When HCMV infects cells in the G0 or G1 phase of the cell cycle, IE1/2 gene expression initiates within 30 minutes, and cell cycle progression is arrested at the G1/S border. In contrast, during S-phase infections, cell cycle progression continues and IE19 is expressed within 6 hours, but IE1/2 expression is delayed until the infected cell passes through mitosis (~16 hpi) and enters the subsequent G1 phase. Image created with BioRender. (**B**) The HCMV UL123 gene (top) contains four exons. Exon 2 contains the start codon (not shown) and the nuclear localization signal (NLS). Exon 4 contains an acidic domain (AD), and a chromatin tethering domain that includes a nucleosome-binding motif (NBM). The canonical RNA (middle) produces a 72 kDa, 489 amino acid IE1 protein. A differentially spliced mRNA (bottom) resulting in an in-frame deletion of the initial segment of exon 4 encodes the 19 kDa, 172 amino acid IE19 protein.

The only published example of an HCMV infection surviving mitosis is the lytic infection of fibroblasts synchronized in the S phase of the cell cycle (23). When fibroblasts in the G0/G1 phase of the cell cycle are infected with HCMV, transcription of IE1 and another driver of lytic infection, IE2, is rapidly initiated (e.g., within 30 minutes; Fig. 1A), and cell cycle progression is arrested at the G1/S border (24). When fibroblasts in the S phase are infected, the cell cycle is initially unperturbed, but IE1 and IE2 transcription does not commence until the infected cell traverses the cell cycle, passes through mitosis, and enters G1 (23) (Fig. 1A). However, upon G1 entry after mitosis, the same percentage of S phase-infected cells initiates IE1 and IE2 transcription as do cells infected during the G1 phase (23, 25), indicating that viral genomes from S-phase infections survive mitosis and are in the nucleus, competent for transcription. Viruses lacking the UL123 CTD show reduced viral gene expression, genome levels, and infectious virion output after S-phase infection and passage through mitosis into the next G1 phase (25), indicating the UL123 CTD is required for S-phase infections.

Interestingly, the canonical CTD-containing UL123 transcript encoding IE1 does not accumulate during S-phase infections (23, 26, 27), but a splice variant (28) encoding the related IE19 protein is expressed (25). The HCMV UL123 gene (Fig. 1B) has four exons. The ORF initiates in exon 2 and the amino-terminal 24 amino acids encoded by exon 2 contain a nuclear localization signal (NLS) (17). A differential splice (Fig. 1B) removing the beginning of exon 4 but then continuing in-frame generates IE19, a 19 kDa protein containing the CTD. IE19 localizes to the nucleus, associates with mitotic chromosomes in a CTD-dependent manner (25), and anomalously migrates in SDS-PAGE at 38 kDa (28). A viral mutant with a silent mutation in the 3′ splice acceptor site of IE19 named WTSS (WT coding sequence, splice site mutation) fails to make IE19 mRNA or protein (29). Identical to the CTD-deletion virus, the WTSS virus shows reduced viral gene expression, genome levels, and infectious virion output after S-phase infection and passage through mitosis into the next G1 phase (25). Exogenously expressed IE19, but not IE1, complements the CTD deletion and WTSS mutant virus defects in S-phase infection (25). Thus, like the CTD, IE19 is required for S-phase infections.

Here, we probe the mechanisms through which IE19 supports S-phase infection. We define the nucleosome-binding motif (NBM) (20) within the CTD as required for S-phase infection. In addition to the CTD/NBM, we identify three lysine residues within exon 2 of IE19 required for S-phase infection. Our work further defines the anatomy of a novel and unique viral tethering protein.

## RESULTS

### The NBM within IE19 is required for viral genome maintenance during S-phase infections

**Fig 2:**
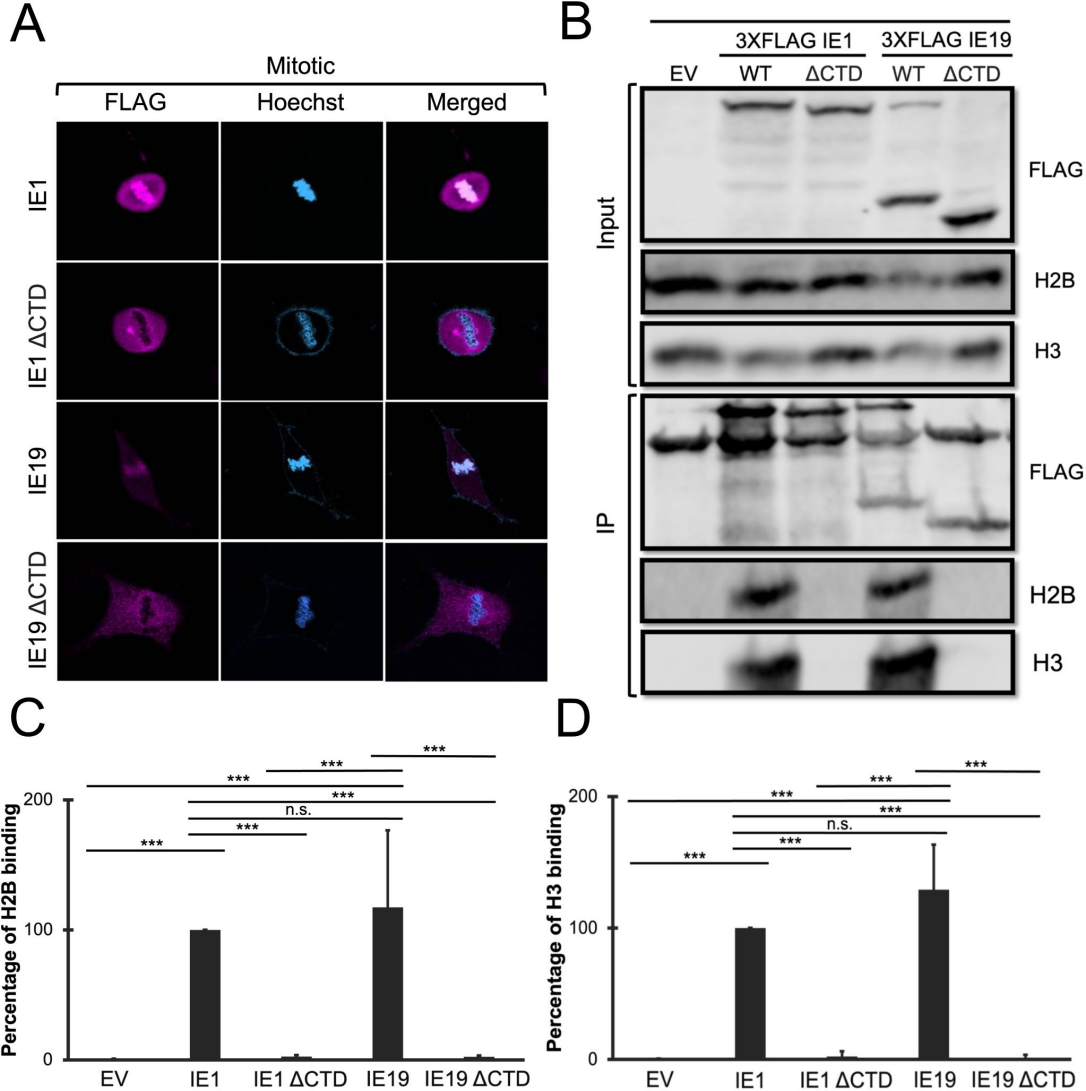
IE19 associates with cellular metaphase chromosomes and binds nucleosomes in a CTD-dependent manner. (**A**) Normal human dermal fibroblasts stably expressing the indicated FLAG-tagged proteins were synchronized with aphidicolin for 24 hours, released for 12 hours, then fixed and stained with a FLAG antibody and Hoechst before visualization by fluorescence microscopy. Representative images of three biological replicates (*n* = 3) are presented. (**B**) Lysates from HEK293T (293) cells transfected with plasmids encoding the indicated FLAG-tagged viral proteins or with an empty vector (EV) control for 48 hours were mixed with nucleosomes extracted from enzymatically digested 293 cell nuclei and then immunoprecipitated with an anti-FLAG antibody. Bound and input samples were separated by SDS-PAGE and analyzed by Western blotting with the indicated antibodies. H2B blots were stripped and then re-probed for H3. Representative images (*n* = 3) are shown. (**C**) Bound histone H2B (means) quantitated from panel B experiments (*n* = 3) are plotted for each condition relative to bound H2B for IE1 (set to 100%). Error bars indicate standard deviations. Results were statistically analyzed by Student’s *t*-test; ***P < 0.001; n.s., not significant (*P* ≥ 0.05). (**D**) Analysis of bound histone H3 as in panel C.

The CTD is found in both the IE1 and IE19 protein products of the UL123 gene. As we previously reported (25), both IE1 and IE19 associate with condensed, mitotic chromosomes aligned at the metaphase plate in a CTD-dependent manner (Fig. 2A) when constitutively expressed in normal human dermal fibroblasts (NHDFs). A NBM responsible for associating with the acidic pocket between histones H2A and H2B within nucleosomes has been mapped to the CTD and shown to function both as an isolated domain and in the context of the IE1 protein (20, 21). We hypothesized that the NBM would also function in the context of IE19 and that it would be required for viral genome maintenance during S-phase infections. To test these hypotheses, we first determined if, like IE1, IE19 interacted with nucleosomes in a CTD-dependent manner. In the established nucleosome-binding assay (20), lysates prepared from 293T cells transfected to express wild-type (WT) or mutant FLAG-tagged IE1 or IE19 proteins were incubated with nucleosomes purified from 293T cells, complexes were collected with FLAG antibodies, and bound histones were identified by Western blot. We confirmed that IE1 associated with histones H2B and H3 in a CTD-dependent manner (20) and determined that IE19 also associated with histones H2B and H3 in a CTD-dependent manner (Fig. 2B). Quantitation of the blots determined that both IE1 and IE19 associated with H2B (Fig. 2C) and H3 (Fig. 2D) in a statistically significant manner. With this assay, no differences between IE1 and IE19 binding to either histone could be distinguished. We conclude that IE19 associates with histones H2B and H3 in a CTD-dependent manner.

**Fig 3:**
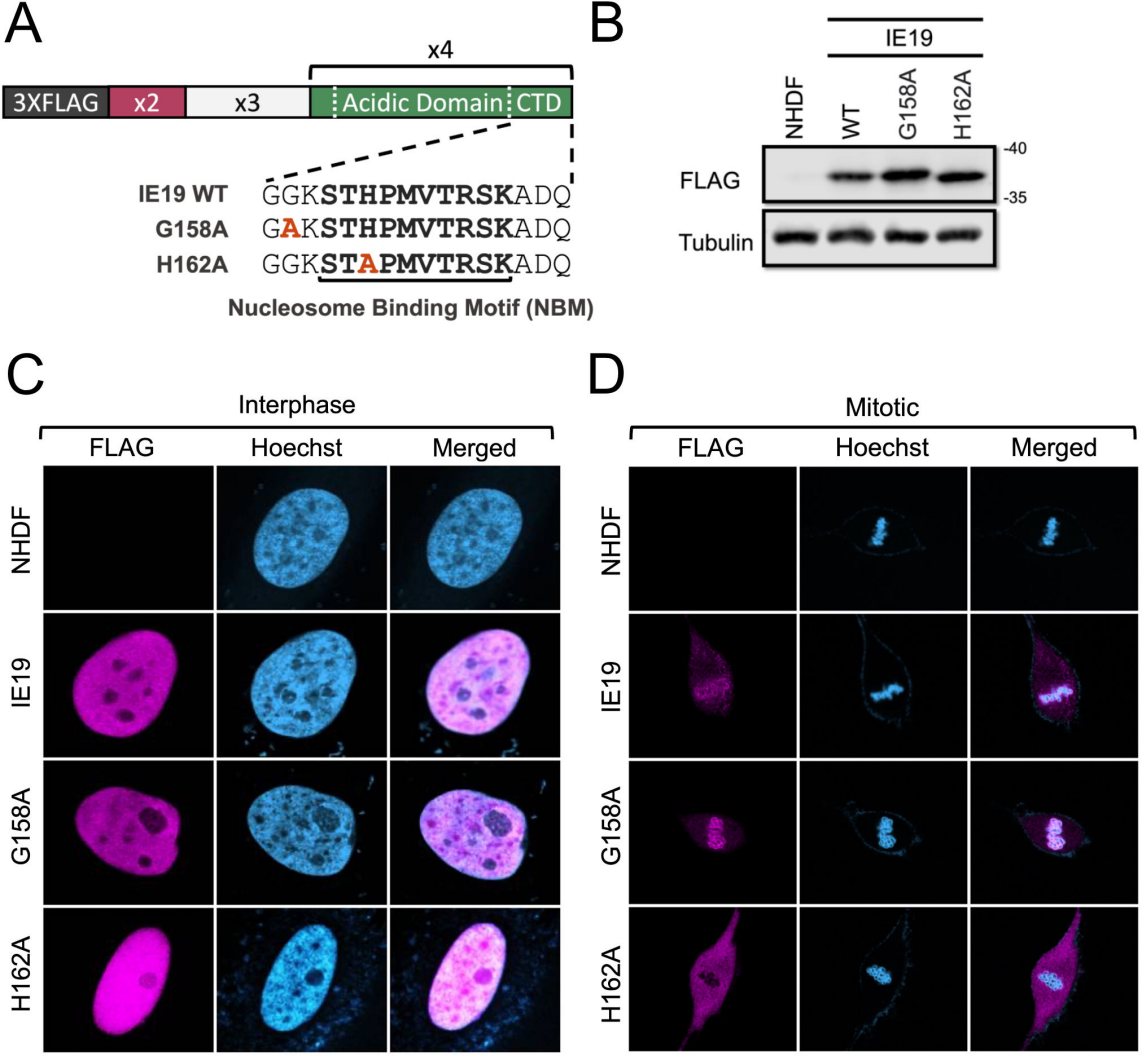
The IE19 nucleosome-binding motif mediates colocalization with mitotic DNA. (**A**) Schematic of 3×FLAG IE19 wild-type showing exon 2 (X2), exon 3 (X3), N-terminally truncated exon 4 (X4), the acidic domain, the chromatin tethering domain, and the nucleosome-binding domain in bolded sequence and indicated by a bracket. Amino acid substitutions in the indicated IE19 mutants are shown in red bold letters. (**B**) Lysates from NHDFs stably expressing the indicated proteins were analyzed by Western blot with the indicated antibodies. Approximate molecular weights are shown. Representative images (*n* = 3) are shown. (**C**) Asynchronous NHDFs stably expressing the indicated proteins were fixed and stained with a FLAG antibody and Hoechst before visualization by fluorescence microscopy. Representative images (*n* = 3) are presented. (**D**) NHDFs stably expressing the indicated FLAG-tagged proteins were synchronized with aphidicolin for 24 hours, released for 12 hours, and then fixed and stained with an anti-FLAG antibody and Hoechst before visualization by fluorescence microscopy. Representative images (*n* = 3) are presented.

**Fig 4:**
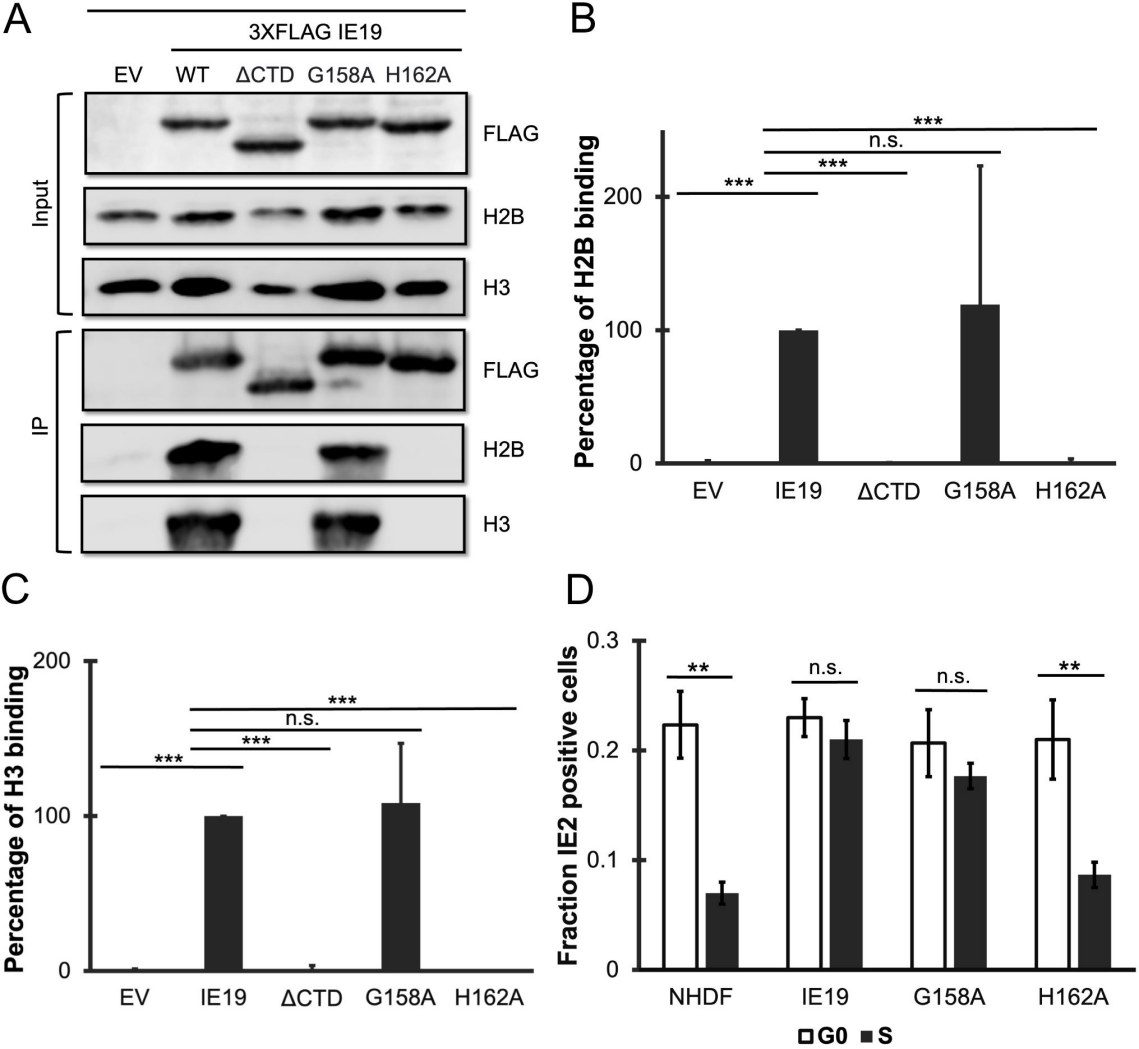
The IE19 NBM is required for successful S-phase infections. (**A**) Lysates from 293s transfected with an EV or plasmids encoding the indicated FLAG-tagged viral proteins for 48 hours were mixed with nucleosome particles extracted from enzymatically digested 293 cell nuclei and then immunoprecipitated with an anti-FLAG antibody. Bound and input samples were separated by SDS-PAGE and subjected to Western blotting with the indicated antibodies. H2B blots were stripped and then re-probed for H3. Representative images (*n* = 3) are shown. (**B**) Bound histone H2B (means) quantitated from panel A experiments (*n* = 3) are plotted for each condition relative to bound H2B for WT IE19 (set to 100%). Error bars indicate standard deviations. Results were statistically analyzed by Student’s *t*-test; ***P < 0.001; n.s., not significant (*P* ≥ 0.05). (**C**) Analysis of bound histone H3 as in panel B. (**D**) NHDFs stably expressing the indicated proteins and synchronized in G0 (open bars) or S phase (filled bars) were infected at an MOI of 0.5 with a CTD-deficient virus (HCMV-ΔCTD). The fraction of IE2-positive cells at 24 hpi was determined by immunofluorescence microscopy and manual counting of at least 500 nuclei per sample and is plotted as the mean from three biological replicates (*n* = 3). Error bars indicate standard deviations. Results were statistically analyzed by Student’s *t*-test; **P < 0.01; ***P < 0.001; and n.s., not significant (*P* ≥ 0.05).

We next asked if the NBM within the CTD was functional in the context of the IE19 protein as it is within IE1. Based on previous mapping (20), we engineered point mutations in IE19 predicted to either maintain (G158A) or eliminate (H162A) nucleosome binding (Fig. 3A) and generated NHDFs that constitutively express each protein (Fig. 3B). Neither mutation impaired the nuclear localization of IE19 in interphase cells (Fig. 3C), but as predicted, the H162A mutation disrupted IE19 association with mitotic chromosomes (Fig. 3D). Similarly, the G158A mutant retained the ability to bind histones (Fig. 4A) H2B (Fig. 4B) and H3 (Fig. 4C), while the H162A mutation eliminated histone binding. We conclude the NBM found in the UL123 CTD functions in the context of IE19.

To determine if the IE19 NBM was required for viral genome maintenance during S-phase infections, we compared the ability of WT, G158A, and H162A IE19 proteins to complement the defect in IE2 protein accumulation observed after S-phase infection of UL123 CTD-deleted (ΔCTD) HCMV with the now standard assay (23, 25). Fewer NHDF cells infected with HCMV-ΔCTD during the S phase of the cell cycle express IE2 at 24 hours than G0 phase cells infected in the same manner (Fig. 4D). Not surprisingly, we found that NHDFs constitutively expressing either WT or NBM-proficient G158A IE19 complemented this defect, but cells expressing the H162A IE19 protein with a crippled NBM did not (Fig. 4D). We conclude that the IE19 NBM is required for viral genome maintenance through mitosis during S-phase infections.

### Sequence features that differentiate IE19 from IE1 do not explain the unique ability of IE19 to support S-phase infections

**Fig 5:**
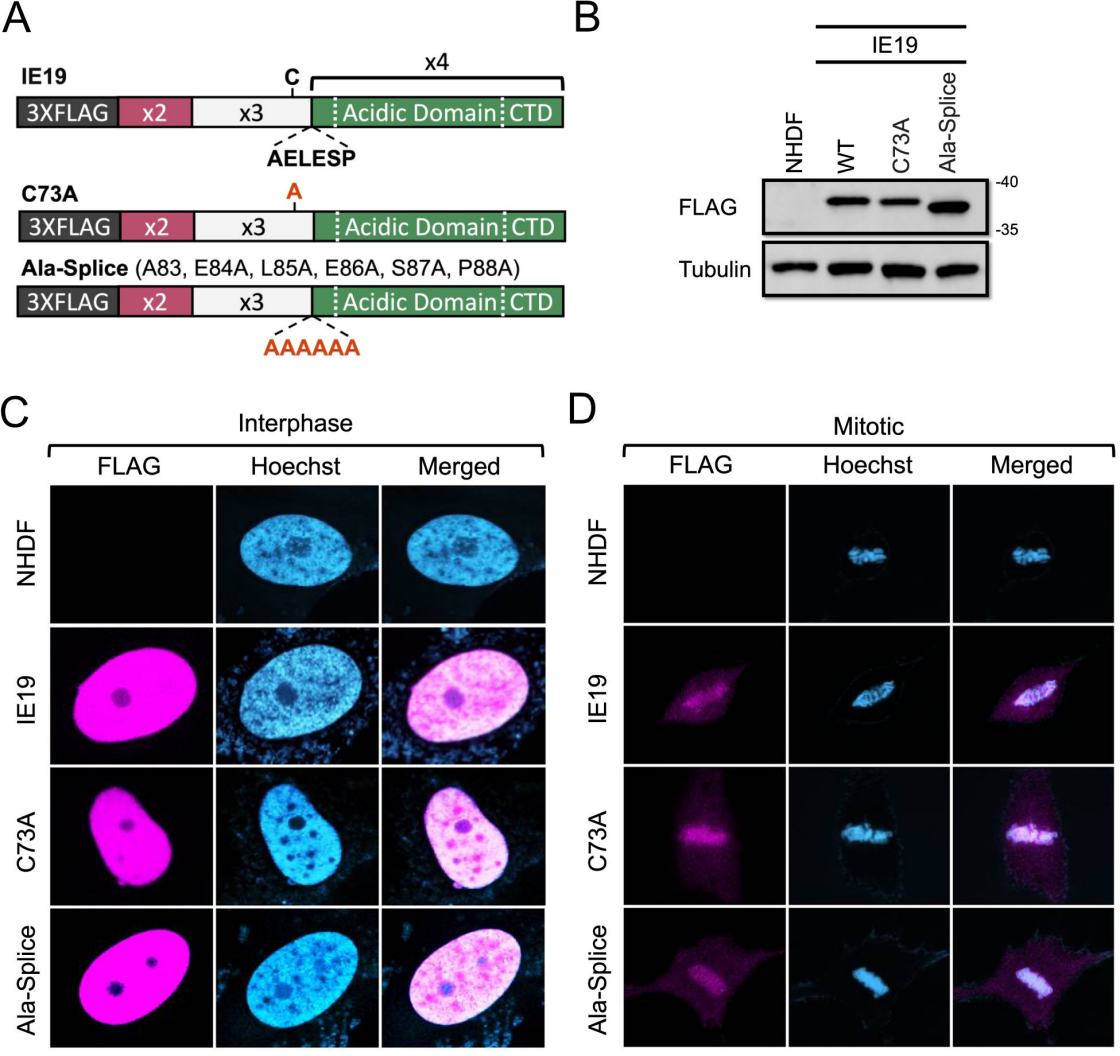
Mutations in the single cysteine residue or at the novel splice junction do not disrupt IE19’s association with mitotic DNA. (**A**) Schematic of 3×FLAG IE19 showing the single cysteine residue (**C**) and the unique amino acid sequence (AELESP) at the novel splice junction in bold. Amino acid substitutions in the indicated IE19 mutants are shown in red bold letters. (**B**) Lysates from NHDFs stably expressing the indicated proteins were analyzed by Western blot with the indicated antibodies. Approximate molecular weights are shown. Representative images (*n* = 3) are shown. (**C**) Asynchronous NHDFs stably expressing the indicated proteins were fixed and stained with a FLAG antibody and Hoechst before visualization by fluorescence microscopy. Representative images (*n* = 3) are presented. (**D**) NHDFs stably expressing the indicated FLAG-tagged proteins were synchronized with aphidicolin for 24 hours, released for 12 hours, and then fixed and stained with an anti-FLAG antibody and Hoechst before visualization by fluorescence microscopy. Representative images (*n* = 3) are presented.

**Fig 6:**
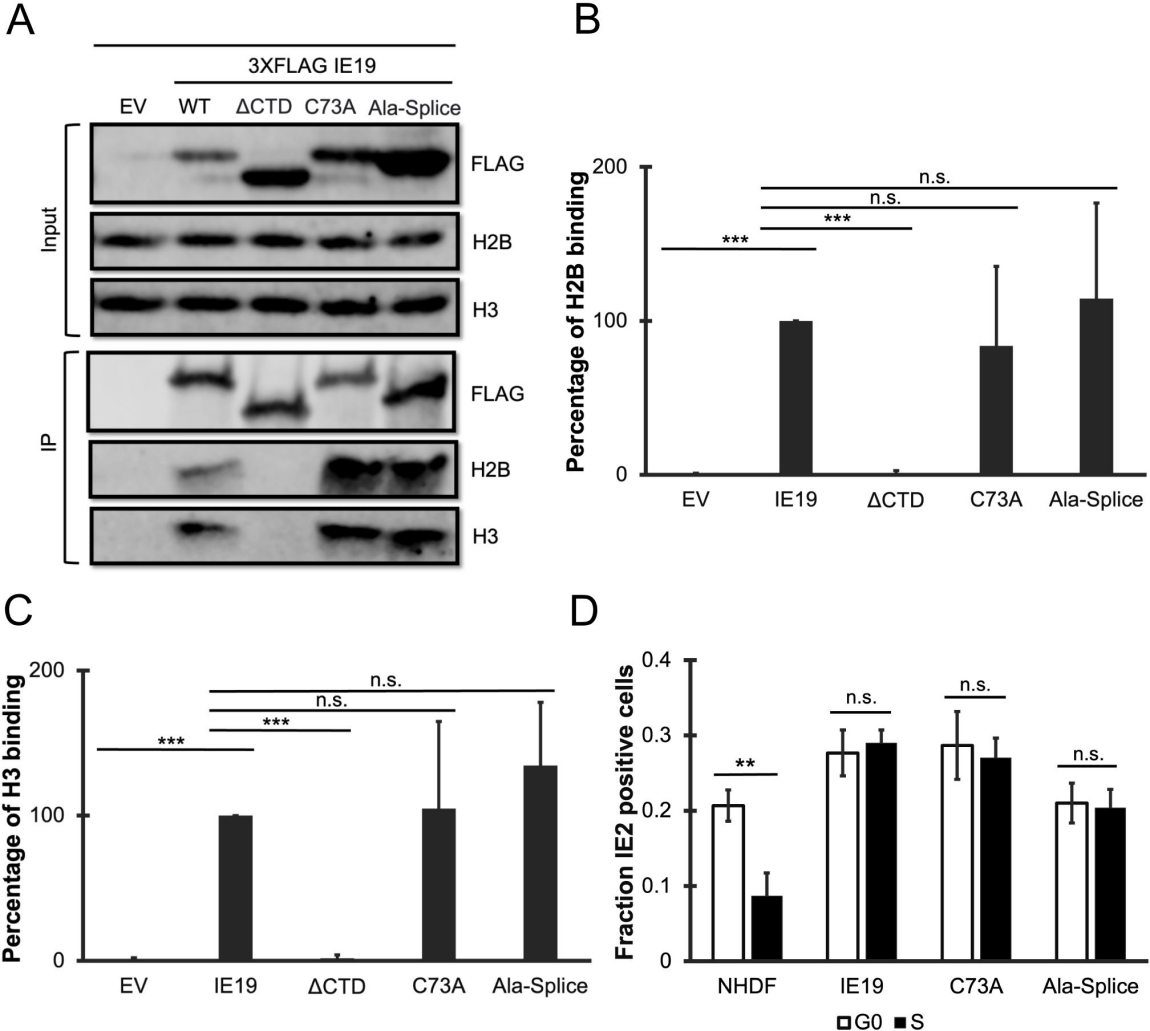
The single cysteine residue or the novel splice junction sequence of IE19 is not required for nucleosome binding or successful S-phase infections. (**A**) Lysates from 293s transfected with an EV or plasmids encoding the indicated FLAG-tagged viral proteins for 48 hours were mixed with nucleosome particles extracted from enzymatically digested 293 cell nuclei and then immunoprecipitated with an anti-FLAG antibody. Bound and input samples were separated by SDS-PAGE and analyzed by Western blotting with the indicated antibodies. H2B blots were stripped and then re-probed for H3. Representative images (*n* = 3) are shown. (**B**) Bound histone H2B (means) quantitated from panel A experiments (*n* = 3) are plotted for each condition relative to bound H2B for WT IE19 (set to 100%). Error bars indicate standard deviations. Results were statistically analyzed by Student’s *t*-test; ***P < 0.001; n.s., not significant (*P* ≥ 0.05). (**C**) Analysis of bound histone H3 as in panel B. (**D**) NHDFs stably expressing the indicated proteins and synchronized in G0 (open bars) or S phase (filled bars) were infected with HCMV-ΔCTD (MOI = 0.5). The fraction of IE2-positive cells at 24 hpi was determined by immunofluorescence microscopy and manual counting of at least 500 nuclei per sample and the mean (*n* = 3) is plotted. Error bars indicate standard deviations. Results were statistically analyzed by Student’s *t*-test; **P < 0.01; ***P < 0.001; and n.s., not significant (*P* ≥ 0.05).

Despite the nucleosome-binding capability conserved between IE1 and IE19 (Fig. 2 to 4), only IE19 supports S-phase infections (25), likely indicating a structural or sequence difference between IE1 and IE19 differentiates this function. The structure of the core domain of IE1 (30, 31) revealed an elongated dimerization domain. Much of the sequence responsible for IE1 dimerization is spliced out of the IE19 transcript and therefore is missing in the IE19 protein (28, 30, 32, 33). Even in the absence of the 319 amino acids that contribute to the extended dimerization domain, IE19 could still potentially dimerize through a disulfide linkage mediated by the single cysteine residue found at amino acid position 73 within the protein (Fig. 5A). Because dimerization or oligomerization is important for the function of other viral tethers (3, 4, 8–13), we asked if IE19 C73 was required for the protein to support S-phase infections. We generated NHDFs that constitutively express an IE19 C73A point mutant (Fig. 5B) that should disrupt any putative disulfide-mediated dimers. We found the C73A mutant protein localized to the nucleus in interphase cells (Fig. 5C), associated with mitotic chromosomes (Fig. 5D) and bound histones (Fig. 6A) H2B (Fig. 6B) and H3 (Fig. 6C) similar to WT IE19. The C73A mutant also complemented the IE2 accumulation defect of HCMV-ΔCTD after S-phase infections (Fig. 6D). We conclude that cysteine 73 is not required for IE19 to support S-phase infections.

The generation of IE19 creates a novel amino acid juxtaposition (compared to IE1) at the splice junction. Therefore, we asked whether the unique amino acid sequence at the splice junction was required for IE19 to support S-phase infections. We generated NHDFs that constitutively express an IE19 mutant (Fig. 5A and B) in which five codons surrounding the splice junction were converted to those encoding alanine, creating a mutant with a run of six consecutive alanines (residues 83–88). We found this Ala-splice mutant protein localized to the nucleus in interphase cells (Fig. 5C), associated with mitotic chromosomes (Fig. 5D) and bound histones (Fig. 6A) H2B (Fig. 6B) and H3 (Fig. 6C) similar to WT IE19. The Ala-splice mutant also complemented the IE2 accumulation defect of HCMV-ΔCTD after S-phase infections (Fig. 6D). We conclude that the unique amino acid juxtaposition at the IE19 splice site is not required for IE19 to support S-phase infections.

### Amino acids encoded within exon 2 represent a second region of the IE19 protein required for S-phase infections

**Fig 7:**
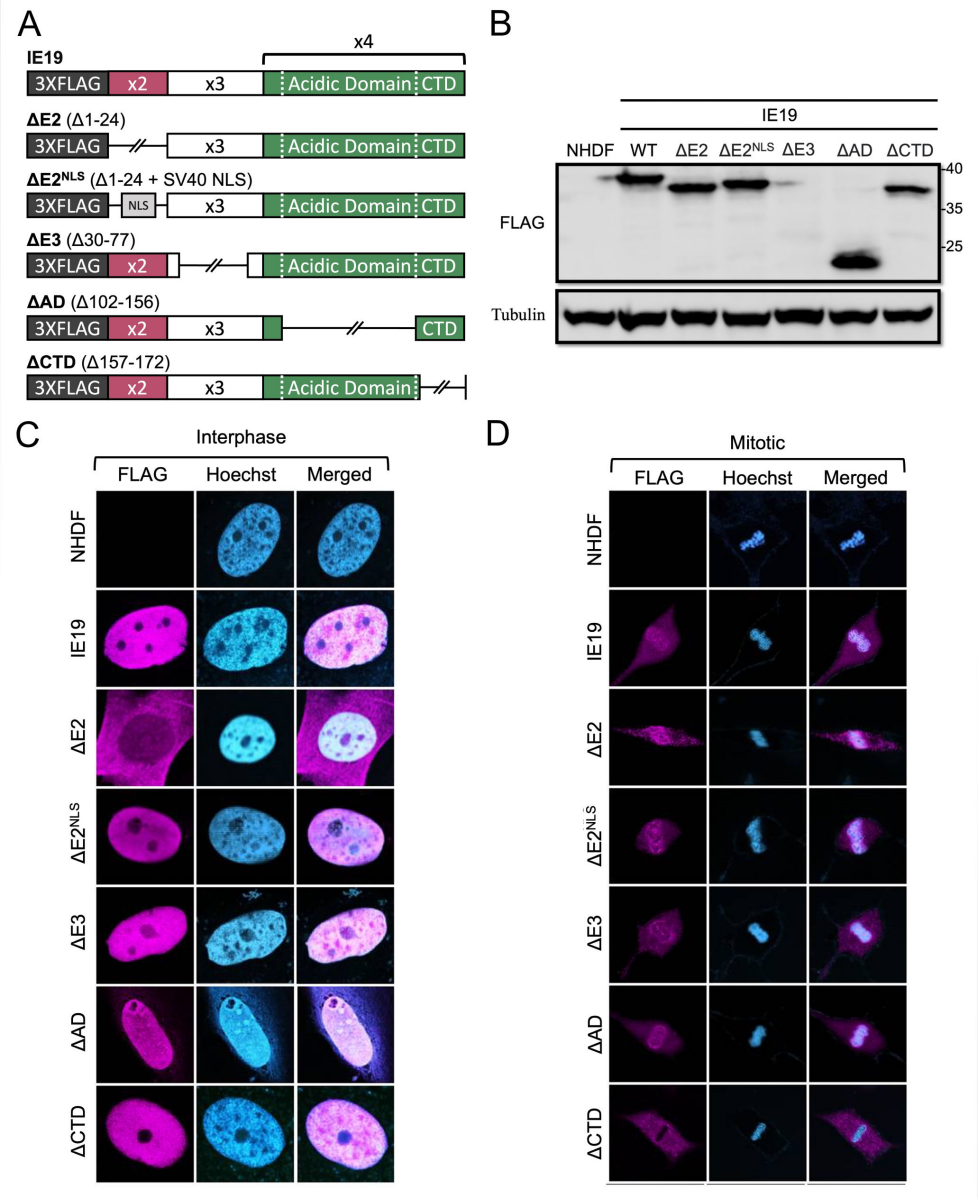
IE19 mutants lacking exon 2, most of exon 3, or acidic domain sequences associate with mitotic chromosomes. (**A**) Schematic of 3×FLAG IE19 showing different deletion mutant alleles and the insertion of a nuclear localization signal. Deleted regions are depicted as broken lines and shown in parentheses as amino acid numbers. (**B**) Lysates from NHDFs stably expressing the indicated proteins were analyzed by Western blot with the indicated antibodies. Approximate molecular weights are shown. Representative images (*n* = 3) are shown. (**C**) Asynchronous NHDFs stably expressing the indicated proteins were fixed and stained with an anti-FLAG antibody and Hoechst before visualization by fluorescence microscopy. Representative images (*n* = 3) are presented. (**D**) NHDFs stably expressing the indicated FLAG-tagged proteins were synchronized with aphidicolin for 24 hours, released for 12 hours, and then fixed and stained with an anti-FLAG antibody and Hoechst before visualization by fluorescence microscopy. Representative images (*n* = 3) are presented.

In addition to a CTD that binds cellular chromosomes, all viral tethers contain additional sequences, located at the other end of the protein, which are responsible for viral genome binding and required for mitotic tethering (3–7). We hypothesized that IE19 sequences in addition to the NBM/CTD would be required for S-phase infections. To test this hypothesis, we generated NHDFs constitutively expressing mutants with deletions in each of the three exons that contribute to IE19 protein sequences (Fig. 7A). We deleted amino acids 1–24 representing all of exon 2 (ΔE2). Because exon 2 contains the NLS, we created an additional mutant in which the exon 2 sequences were replaced with the SV40 NLS (ΔE2^NLS^). We deleted amino acids 30–77 representing most of exon 3 (ΔE3) based on previous work (34) demonstrating this mutation was compatible with viral growth. Finally, we deleted amino acids 102–156 representing the acidic domain of exon 4 (ΔAD). All three exon mutants (ΔE2, ΔE3, and ΔAD) retain the NBM/CTD (Fig. 7A). Our analysis included WT IE19 and IE19 ΔCTD as positive and negative controls, respectively.

The mutants accumulated to steady-state levels on Western blots similar to the WT protein except for ΔE3 (Fig. 7B). During mutant virus infections, IE1 and IE2 proteins with exon 3 deletions also accumulated to lower steady-state levels (34). It is currently unclear whether the lower accumulation of exon 3 mutant proteins results from decreased stability, decreased transactivation ability, or some combination of both. The ΔAD protein migrated with an apparent molecular weight that more accurately approximated the predicted molecular weight based on amino acid sequence (Fig. 7B). Thus, it appears the acidic nature of IE19 imparted by the acidic domain is largely responsible for the aberrant migration of the protein in SDS-PAGE, as we previously speculated (25).

**Fig 8:**
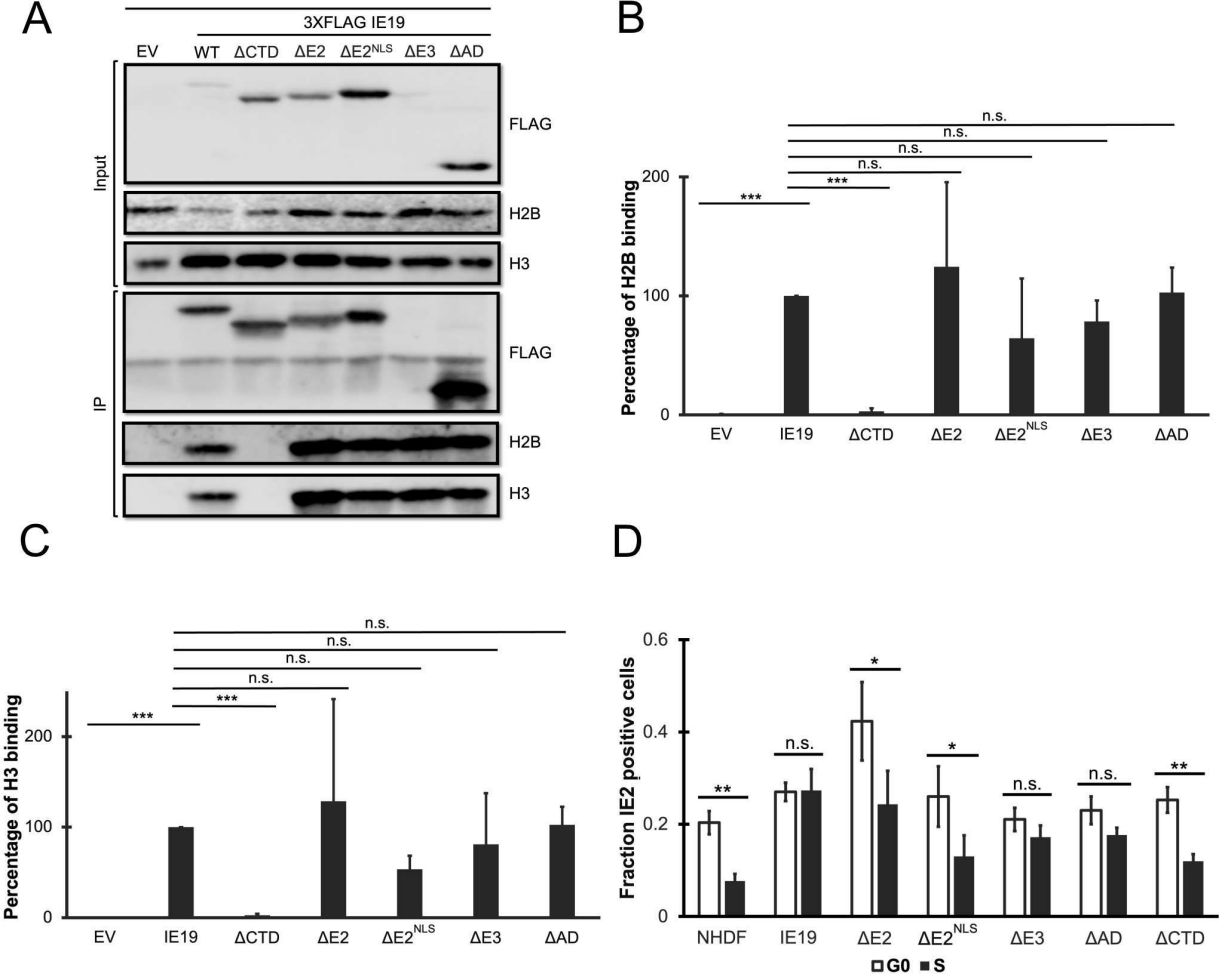
IE19 exon 2 is not required for nucleosome binding but is required for successful S-phase infections. (**A**) Lysates from 293s transfected with an EV or plasmids encoding the indicated FLAG-tagged viral proteins for 48 hours were mixed with nucleosomes extracted from enzymatically digested 293 cell nuclei and then immunoprecipitated with an anti-FLAG antibody. Bound and input samples were separated by SDS-PAGE and analyzed by Western blotting with the indicated antibodies. H2B blots were stripped and then re-probed for H3. Representative images (*n* = 3) are shown. (**B**) Bound histone H2B (means) quantitated from panel A experiments (*n* = 3) are plotted for each condition relative to bound H2B for WT IE19 (set to 100%). Error bars indicate standard deviations. Results were statistically analyzed by Student’s *t*-test; ***P < 0.001; n.s., not significant (*P* ≥ 0.05). (**C**) Analysis of bound histone H3 as in panel B. (**D**) NHDFs stably expressing the indicated proteins and synchronized in G0 (open bars) or S phase (filled bars) were infected with HCMV-ΔCTD (MOI = 0.5). The fraction of IE2-positive cells at 24 hpi was determined by immunofluorescence microscopy and manual counting of at least 500 nuclei per sample and the mean (*n* = 3) is plotted. Error bars indicate standard deviations. Results were statistically analyzed by Student’s *t*-test; *P < 0.05; **P < 0.01; ***P < 0.001; and n.s., not significant (*P* ≥ 0.05).

The mutants localized to the nucleus in interphase cells except for ΔE2 (Fig. 7C), as expected due to the absence of an NLS in this mutant protein. Furthermore, all the mutants, including ΔE2, associated with mitotic chromosomes (Fig. 7D). As expected, ΔCTD did not. It is likely that ΔE2 can associate with mitotic chromosomes despite lacking an NLS because the nuclear envelope breakdown coincident with mitosis ends the physical separation into distinct subcellular compartments of cytoplasmic IE19 ΔE2 and nuclear cellular chromosomes. Despite the reduced detection of IE19 ΔE3 on Western blots, we could readily detect it by indirect immunofluorescence microscopy (Fig. 7C and D), indicating the protein in cells (or in fixed cells) might be more stable or better recognized by antibodies compared to the protein in cellular lysates. The mutants all bound histones (Fig. 8A) H2B (Fig. 8B) and H3 (Fig. 8C) similar to WT IE19. Importantly, mutants lacking exon 3 or exon 4 sequences remained fully capable of supporting S-phase infections, whereas both mutants lacking exon 2 sequences (ΔE2 and ΔE2^NLS^) failed to complement the IE2 accumulation defect of HCMV-ΔCTD after S-phase infections (Fig. 8D). We conclude that exon 2 represents a second region of IE19 required to support S-phase infections. Because ΔE2^NLS^ localizes to the nucleus and because exon 2 sequences are dispensable for nucleosome binding, we speculate exon 2 sequences may be required to bind the viral genome (see Discussion).

### All three lysine residues encoded by exon 2 of the IE19 protein are required for S-phase infections

**Fig 9:**
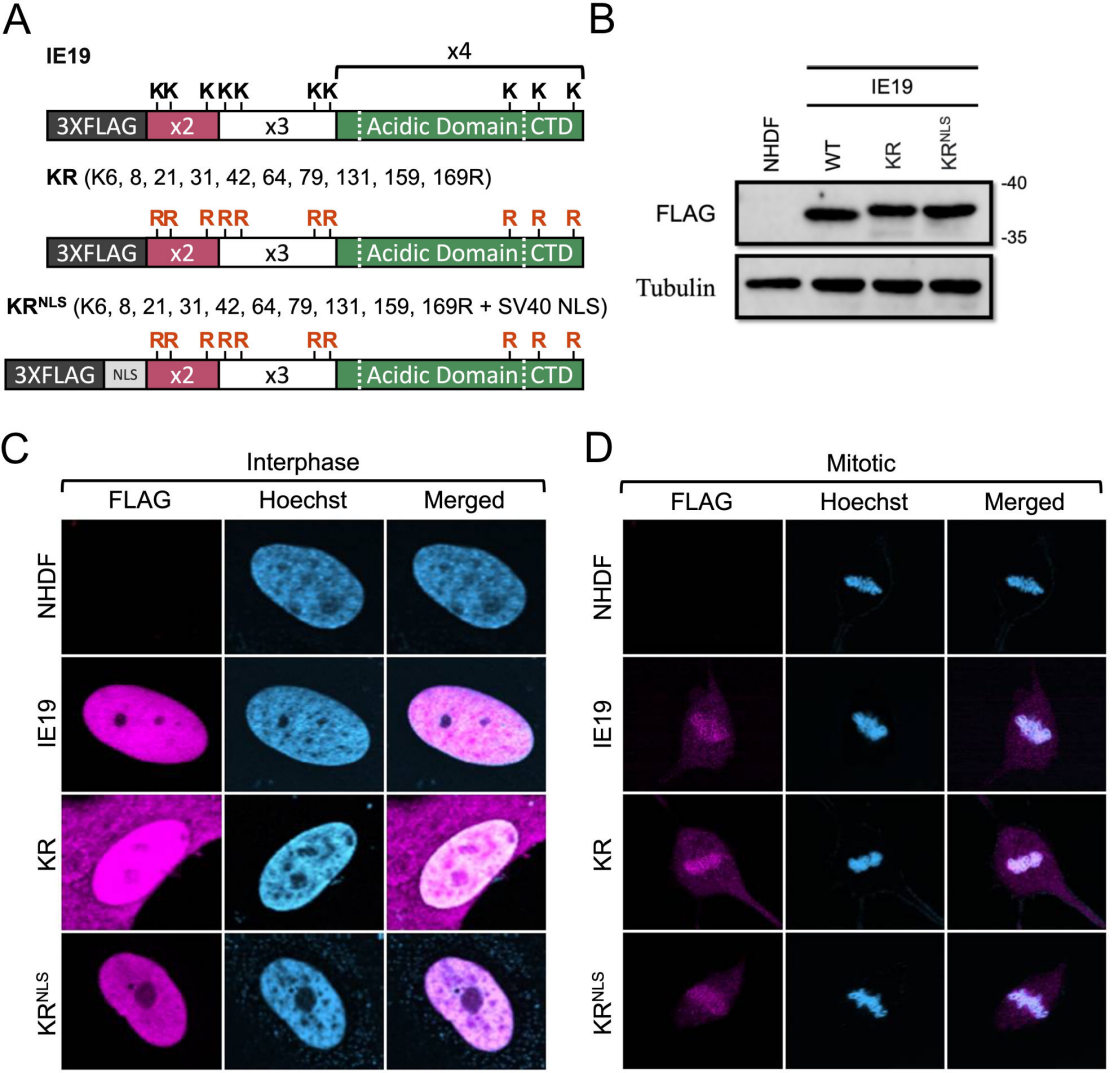
Lysine residues of IE19 contribute to nuclear localization but are not required for mitotic chromosome association. (**A**) Schematic of 3×FLAG IE19 showing a lysine to arginine (KR) substitution mutant without and with an NLS. Mutations are shown in red bold letters. (**B**) Lysates from NHDFs stably expressing the indicated proteins were analyzed by Western blot with the indicated antibodies. Approximate molecular weights are shown. Representative images (*n* = 3) are shown. (**C**) Asynchronous NHDFs stably expressing the indicated proteins were fixed and stained with an anti-FLAG antibody and Hoechst before visualization by fluorescence microscopy. Representative images (*n* = 3) are presented. (**D**) NHDFs stably expressing the indicated FLAG-tagged proteins were synchronized with aphidicolin for 24 hours, released for 12 hours, and then fixed and stained with an anti-FLAG antibody and Hoechst before visualization by fluorescence microscopy. Representative images (*n* = 3) are presented.

Though we can easily detect IE19 after ectopic expression (transient or constitutive), we (25) and others before us (32, 35) have been unable to detect IE19 during HCMV infections. The inability to detect IE19 during infection may stem from low protein expression (transcription, mRNA stability, or translation), low protein stability, or a combination of both. In an attempt to increase the stability of the IE19 protein, we generated an allele in which all 10 lysines within the protein are converted to arginines (KR) (Fig. 9A). Lysines are substrates for the polyubiquitination that leads to the proteasomal degradation of the modified protein (36–38). The conversion of lysines to arginines maintains the positive charge but eliminates all but N-terminal polyubiquitination, which thus often stabilizes proteins and increases their steady-state levels (39, 40). Because lysines also participate in the function of NLSs (41–43), we also generated a lysine-less IE19 allele with an added NLS (KR^NLS^) (Fig. 9A). WT, KR, or KR^NLS^ IE19 proteins accumulated to similar steady-state levels upon constitutive expression in NHDFs (Fig. 9B). Thus, mutation of lysines of IE19 did not appreciably increase protein accumulation, indicating the low levels observed upon infection may result from low expression (or destabilization by another viral or viral induced cellular protein). Despite the lack of effect on protein levels, we completed our characterization of these mutants.

**Fig 10:**
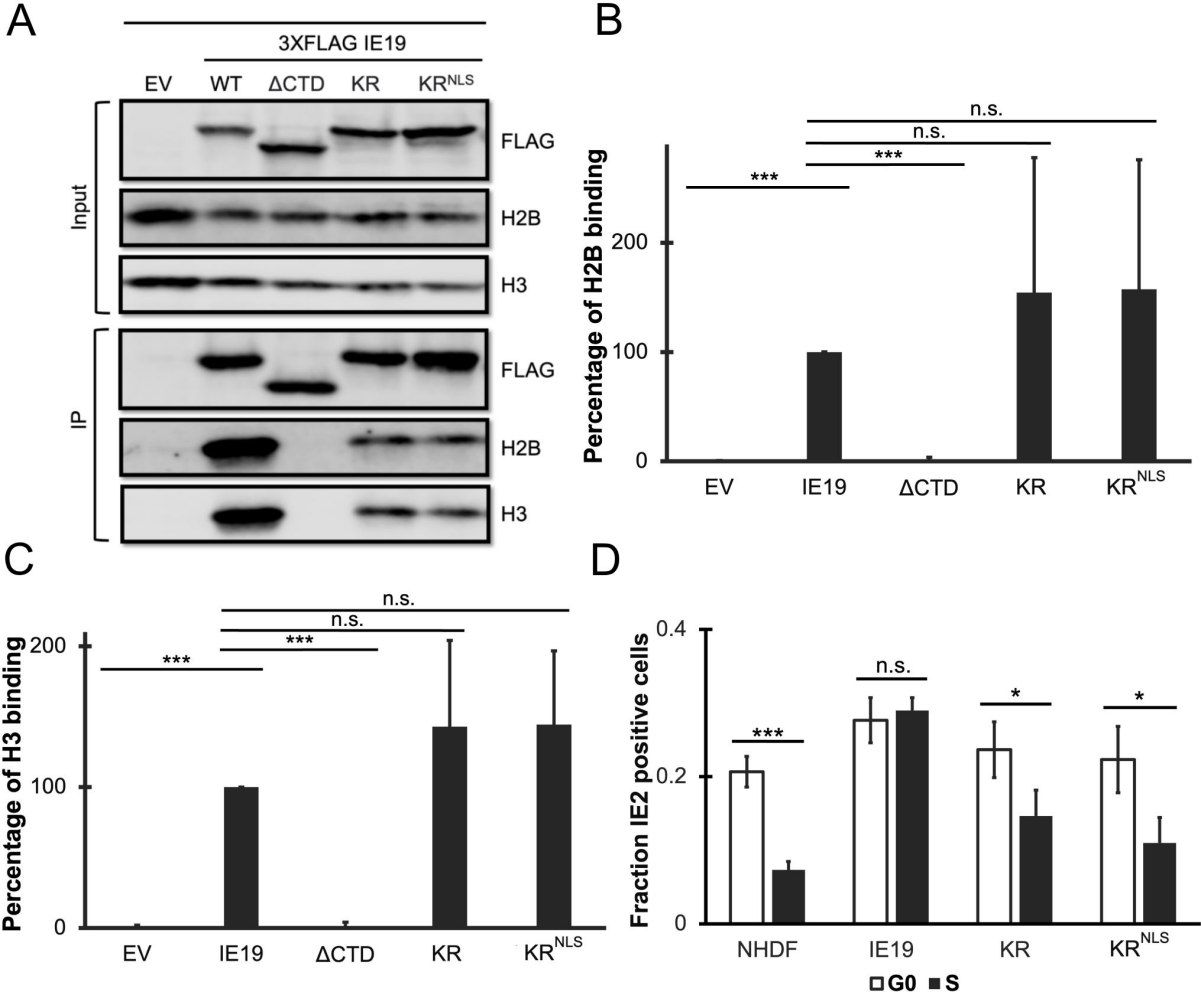
IE19 lysines are dispensable for nucleosome binding but one or more are required for successful S-phase infections. (**A**) Lysates from 293s transfected with an EV or plasmids encoding the indicated FLAG-tagged viral proteins for 48 hours were mixed with nucleosomes extracted from enzymatically digested 293 cell nuclei and then immunoprecipitated with an anti-FLAG antibody. Bound and input samples were separated by SDS-PAGE and analyzed by Western blotting with the indicated antibodies. H2B blots were stripped and then re-probed for H3. Representative images (*n* = 3) are shown. (**B**) Bound histone H2B (means) quantitated from panel A experiments (*n* = 3) are plotted for each condition relative to bound H2B for WT IE19 (set to 100%). Error bars indicate standard deviations. Results were statistically analyzed by Student’s *t*-test; ***P < 0.001; n.s., not significant (*P* ≥ 0.05). (**C**) Analysis of bound histone H3 as in panel B. (**D**) NHDFs stably expressing the indicated proteins and synchronized in G0 (open bars) or S phase (filled bars) were infected with HCMV-ΔCTD (MOI = 0.5). The fraction of IE2-positive cells at 24 hpi was determined by immunofluorescence microscopy and manual counting of at least 500 nuclei per sample and the mean (*n* = 3) is plotted. Error bars indicate standard deviations. Results were statistically analyzed by Student’s *t*-test; *P < 0.05; **P < 0.01; ***P < 0.001; and n.s., not significant (*P* ≥ 0.05).

Both mutant proteins localized to the nucleus in interphase cells (Fig. 9C), although the KR mutant also showed some cytoplasmic staining. Both mutant proteins associated with mitotic chromosomes (Fig. 9D) and bound histones (Fig. 10A) H2B (Fig. 10B) and H3 (Fig. 10C) similar to WT IE19. Provocatively, neither mutant was able to complement the IE2 accumulation defect of HCMV-ΔCTD after S-phase infections (Fig. 10D). We conclude that one or more lysine residues within IE19 are required for IE19 to support S-phase infections.

**Fig 11:**
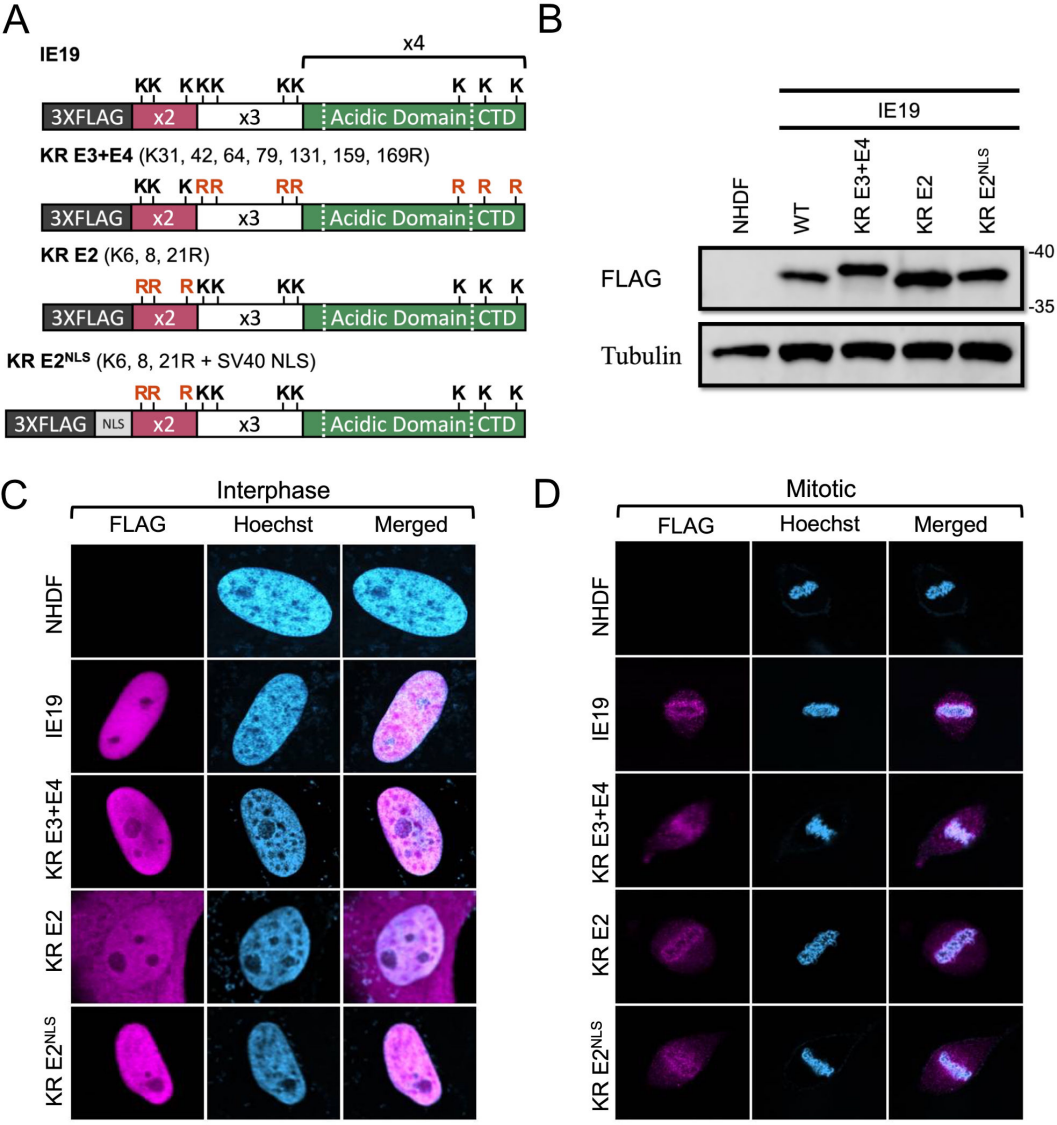
Lysine residues in exon 2 of IE19 contribute to nuclear localization but are not required for mitotic chromosome association. (**A**) Schematic of 3×FLAG IE19 showing different lysine to arginine (KR) substitution mutants without or with an NLS. Mutations are shown in red bold letters. (**B**) Lysates from NHDFs stably expressing the indicated proteins were analyzed by Western blot with the indicated antibodies. Approximate molecular weights are shown. Representative images (*n* = 3) are shown. (**C**) Asynchronous NHDFs stably expressing the indicated proteins were fixed and stained with an anti-FLAG antibody and Hoechst before visualization by fluorescence microscopy. Representative images (*n* = 3) are presented. (**D**) NHDFs stably expressing the indicated FLAG-tagged proteins were synchronized with aphidicolin for 24 hours, released for 12 hours, and then fixed and stained with an anti-FLAG antibody and Hoechst before visualization by fluorescence microscopy. Representative images (*n* = 3) are presented.

**Fig 12:**
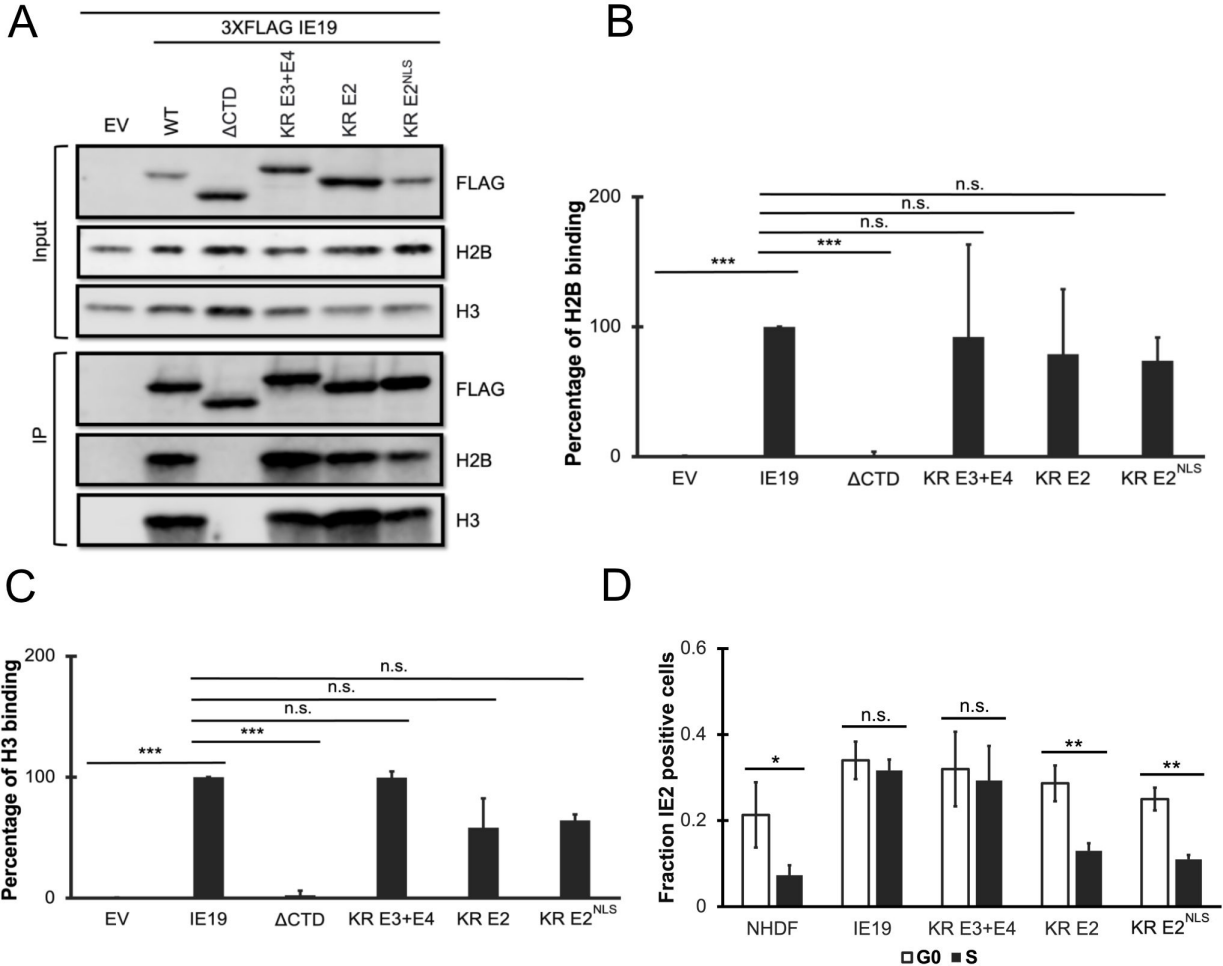
Lysine residues in exon 2 of IE19 are dispensable for nucleosome binding but one or more are required for successful S-phase infections. (**A**) Lysates from 293s transfected with an EV or plasmids encoding the indicated FLAG-tagged viral proteins for 48 hours were mixed with nucleosomes extracted from enzymatically digested 293 cell nuclei and then immunoprecipitated with an anti-FLAG antibody. Bound and input samples were separated by SDS-PAGE and analyzed by Western blotting with the indicated antibodies. H2B blots were stripped and then re-probed for H3. Representative images (*n* = 3) are shown. (**B**) Bound histone H2B (means) quantitated from panel A experiments (*n* = 3) are plotted for each condition relative to bound H2B for WT IE19 (set to 100%). Error bars indicate standard deviations. Results were statistically analyzed by Student’s *t*-test; ***P < 0.001; n.s., not significant (*P* ≥ 0.05). (**C**) Analysis of bound histone H3 as in panel B. (**D**) NHDFs stably expressing the indicated proteins and synchronized in G0 (open bars) or S phase (filled bars) were infected with HCMV-ΔCTD (MOI = 0.5). The fraction of IE2-positive cells at 24 hpi was determined by immunofluorescence microscopy and manual counting of at least 500 nuclei per sample and the mean (*n* = 3) is plotted. Error bars indicate standard deviations. Results were statistically analyzed by Student’s *t*-test; *P < 0.05; **P < 0.01; ***P < 0.001; and n.s., not significant (*P* ≥ 0.05).

Our combined observations that one or more lysines and sequences within exon 2 are required for IE19 to support S-phase infections led us to hypothesize that one or more of the lysines within exon 2 are required for IE19 to support S-phase infections. To test this hypothesis, we generated NHDFs constitutively expressing mutants with lysine to arginine substitutions either in only exon 2 without (KR E2) or with (KR E2^NLS^) an added SV40 NLS or in exons 3 and 4 combined (KR E3 + E4) (Fig. 11A). Each mutant accumulated to steady-state levels on Western blots similar to the WT protein (Fig. 11B) and localized to the nucleus in interphase cells (Fig. 11C), although KR-E2 showed substantial cytoplasmic staining, as predicted. Each mutant also associated with mitotic chromosomes (Fig. 11D) and bound histones (Fig. 12A) H2B (Fig. 12B) and H3 (Fig. 12C) similar to WT IE19. Importantly, the mutant with lysine to arginine substitutions in exon 3 and exon 4 sequences remained fully capable of supporting S-phase infections, whereas both mutants (KR E2 and KR E2^NLS^) with lysine to arginine substitutions in exon 2 sequences failed to complement the IE2 accumulation defect of HCMV-ΔCTD after S-phase infections (Fig. 12D). We conclude that one or more of the lysine residues within exon 2 are required to support S-phase infections.

**Fig 13:**
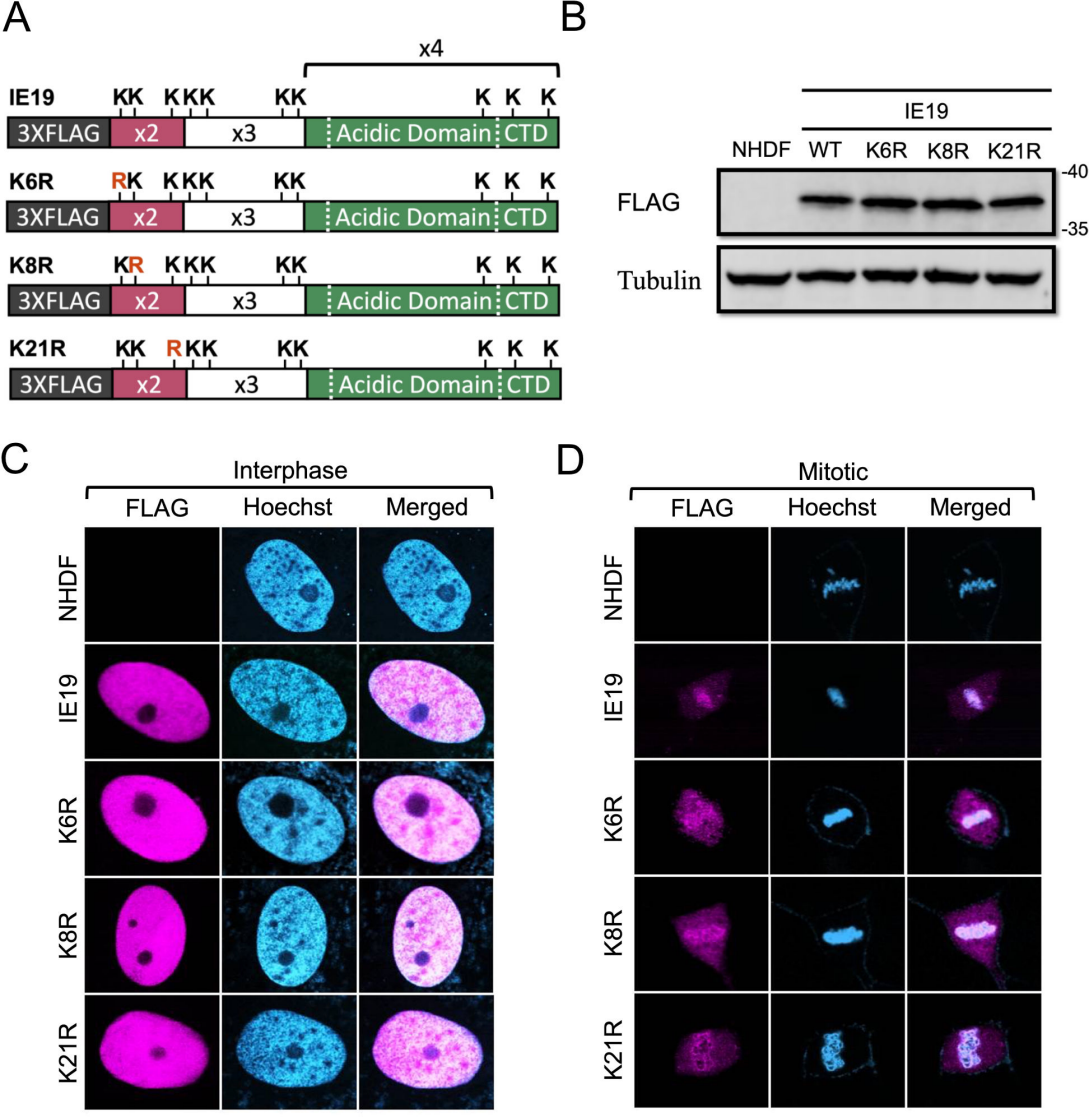
No single lysine in exon 2 is required for IE19 nuclear localization or mitotic chromosome association. (**A**) Schematic of 3×FLAG IE19 showing different lysine to arginine (KR) substitution mutants within exon 2. Mutations are shown in red bold letters. (**B**) Lysates from NHDFs stably expressing the indicated proteins were analyzed by Western blot with the indicated antibodies. Approximate molecular weights are shown. Representative images (*n* = 3) are shown. (**C**) Asynchronous NHDFs stably expressing the indicated proteins were fixed and stained with an anti-FLAG antibody and Hoechst before visualization by fluorescence microscopy. Representative images (*n* = 3) are presented. (**D**) NHDFs stably expressing the indicated FLAG-tagged proteins were synchronized with aphidicolin for 24 hours, released for 12 hours, and then fixed and stained with an anti-FLAG antibody and Hoechst before visualization by fluorescence microscopy. Representative images (*n* = 3) are presented.

**Fig 14:**
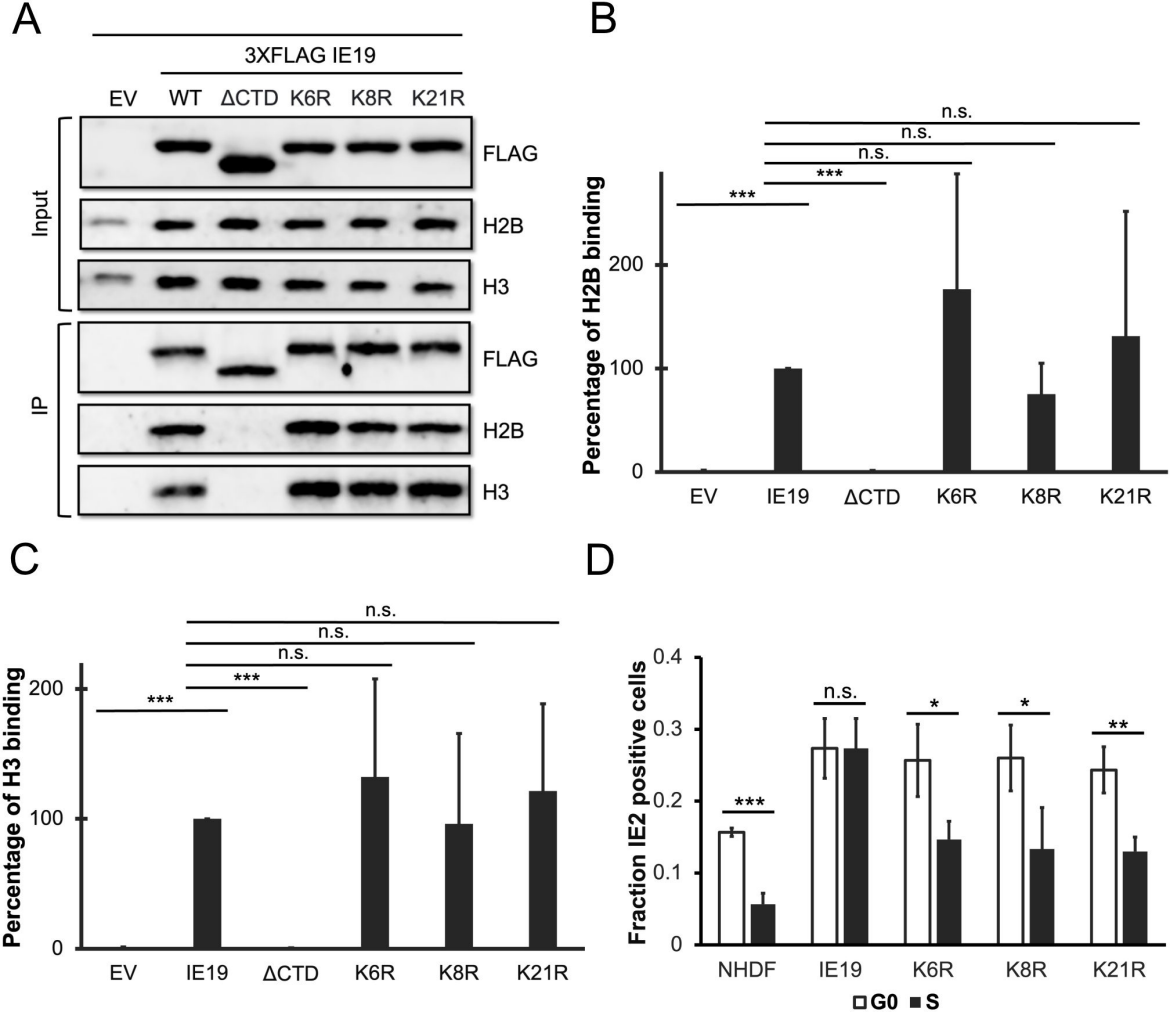
None of the three lysines in IE19 are required for nucleosome binding but each contributes to successful S-phase infections. (**A**) Lysates from 293s transfected with an EV or plasmids encoding the indicated FLAG-tagged viral proteins for 48 hours were mixed with nucleosomes extracted from enzymatically digested 293 cell nuclei and then immunoprecipitated with an anti-FLAG antibody. Bound and input samples were separated by SDS-PAGE and analyzed by Western blotting with the indicated antibodies. H2B blots were stripped and then re-probed for H3. Representative images (*n* = 3) are shown. (**B**) Bound histone H2B (means) quantitated from panel A experiments (*n* = 3) are plotted for each condition relative to bound H2B for WT IE19 (set to 100%). Error bars indicate standard deviations. Results were statistically analyzed by Student’s *t*-test; ***P < 0.001; n.s., not significant (*P* ≥ 0.05). (**C**) Analysis of bound histone H3 as in panel B. (**D**) NHDFs stably expressing the indicated proteins and synchronized in G0 (open bars) or S phase (filled bars) were infected with HCMV-ΔCTD (MOI = 0.5). The fraction of IE2-positive cells at 24 hpi was determined by immunofluorescence microscopy and manual counting of at least 500 nuclei per sample and the mean (*n* = 3) is plotted. Error bars indicate standard deviations. Results were statistically analyzed by Student’s *t*-test; *P < 0.05; **P < 0.01; ***P < 0.001; and n.s., not significant (*P* ≥ 0.05).

**Fig 15:**
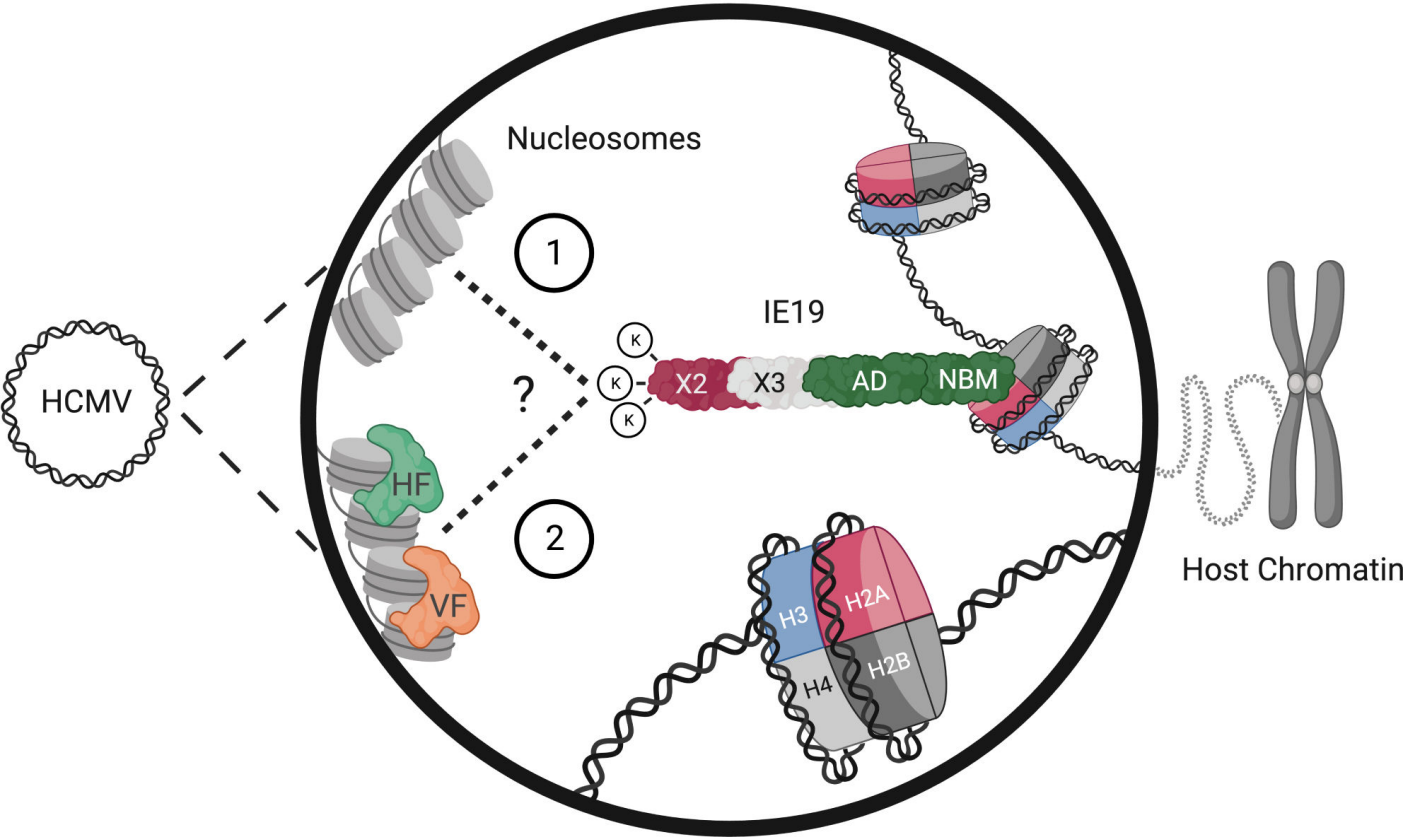
Model for IE19-mediated maintenance of the HCMV genome through mitosis. During mitosis, IE19 is predicted to associate with the viral genome using each of its three lysine residues encoded by exon 2. Viral genome association could be directly with the DNA or through factors encoded by the virus (VF) or the host (HF). IE19 simultaneously attaches to host chromosomes using its NBM within the CTD to bind within the acidic patch between H2A and H2B within nucleosomes on mitotic chromatin. Image created with BioRender.

Finally, to determine which lysine residue(s) within exon 2 are required for IE19 to support S-phase infection, we generated NHDFs constitutively expressing mutants with single lysine to arginine substitutions in exon 2 (K6R, K8R, or K21R) (Fig. 13A). Because we disrupted only a single lysine in each mutant, we did not add an exogenous NLS. Each mutant accumulated to steady-state levels on Western blots similar to the WT protein (Fig. 13B) and localized to the nucleus in interphase cells (Fig. 13C) in the absence of an exogenous NLS. Each mutant also associated with mitotic chromosomes (Fig. 13D) and bound histones (Fig. 14A) H2B (Fig. 14B) and H3 (Fig. 14C) similar to WT IE19. Provocatively, none of the single lysine to arginine substitution mutants in exon 2 could support S-phase infections. Each failed to complement the IE2 accumulation defect of HCMV-ΔCTD after S-phase infections (Fig. 14D). We conclude that each of the three lysine residues within exon 2 is required to support S-phase infections. In total, we conclude that each of the three lysines in exon 2, and the NBM in the CTD within exon 4, are required for IE19 to allow HCMV to infect cells within the S phase of the cell cycle (Fig. 15).

## DISCUSSION

Oncogenic viruses tether their extrachromosomal genomes to cellular chromosomes during persistence to allow them to re-establish nuclear localization after nuclear envelope breakdown during mitosis. Known viral tethers contain an N-terminal CTD that binds cellular chromosomes and a C-terminal domain that binds sequence-specific elements on the viral genome (3–7). We previously demonstrated the HCMV IE19 protein mediates viral genome survival through mitosis after S-phase infections (25). Here, we show that an NBM within a carboxy-terminal CTD (Fig. 3 and 4) and an N-terminal triple lysine motif (Fig. 13 and 14) in IE19 are required for S-phase infections. No function other than nucleosome binding/chromatin tethering has been described for the UL123 CTD, and the NBM constitutes the majority of the CTD, likely indicating that nucleosome binding is the sole function of the UL123 CTD. As in the known viral tethers, the IE19 CTD/NBM is required for association with mitotic chromosomes (Fig. 3). The other viral tethers bind to preferred sites within cellular genomes (44–50) through different mechanisms. HPV E2 associates with the cellular BRD4 protein that binds acetylated histones, EBV EBNA1 uses AT hooks to bind to AT-rich sequences, and KSHV LANA uses a small peptide to bind within the acidic interface of dimerized histones H2A-H2B. Additional chromatin-associated cellular proteins participate in the interaction of these proteins with their respective targets. The CTD/NBM of IE19 interacts with nucleosomes in a manner similar to KSHV LANA (20, 21), and thus these two viral tethers may associate with similar cellular chromosomal sites. Whether IE19 binds to specific, preferred, or random sites within cellular genomes remains to be determined.

We hypothesize that the triple lysine motif in exon 2 near the N terminus of IE19 is responsible for viral genome binding because mutants with disruptions in this motif maintain the ability to bind nucleosomes and interact with mitotic chromosomes but are unable to support S-phase infections (Fig. 13 and 14). In support of this hypothesis, a lysine in the HPV E2 C-terminal DNA-binding domain increases the affinity of the protein for the E2 binding sites repeated within the viral genome (51). If or where IE19 binds to the viral genome remains to be explored. The other viral tethers all bind repeated sequences on viral genomes (52–56). HPV E2 binds to 12 bp motifs in the viral early promoter, EBV EBNA1 binds to 30 bp repeats in the dyad symmetry elements and the family of repeats, and KSHV LANA binds to repeated 18 bp sequences in the terminal repeats. Interestingly, the HCMV terminal repeats have been proposed as a plasmid maintenance element (57), making it possible that IE19 might interact with the viral repeats.

Positively charged lysines can help mediate non-specific interactions of proteins with the negatively charged phosphate backbone of DNA (58–60), and the positive charge of the lysine patch within the viral genome-binding domain of LANA is required for binding to the acidic patch of BET proteins BRD2 and BRD4 (61, 62). However, our arginine substitution data (Fig. 10D, 12D, and 14D) indicate that a positive charge in exon 2 of IE19 is not sufficient for S-phase infections, indicating that a feature of the lysine residues other than their charge is likely required. Unlike arginines, lysines are subject to numerous post-translational modifications (PTMs), including acetylation (63, 64). Acetylated lysines serve as binding sites for bromodomain-containing proteins (65–69), including BRD2 and BRD4. Furthermore, a group of three acetylated lysines increases the affinity of the human immunodeficiency virus integrase protein for viral DNA (70). Thus, it is possible that PTM of the exon 2 lysines within IE19, perhaps acetylations, may be required for its ability to support S-phase infections.

The IE1 protein shares both the exon 2 triple lysine motif and the CTD/NBM with IE19 and associates with mitotic chromosomes (15, 19, 20, 22, 28, 57, 71) but fails to support S-phase infections in the absence of IE19 (25), indicating IE19 is necessary for S-phase infections. Our assays test the function of IE19 to complement S-phase infections of a virus capable of making a truncated (CTD-deleted) IE1, so we cannot say that IE19 is sufficient for S-phase infections, although that is likely the case. We hypothesize that IE1 cannot complement S-phase infections because it might fail to bind the viral genome. The central core of IE1 that is missing from IE19 is a dimerization domain (30, 33). Perhaps, the dimer structure of IE1 orients the two functional motifs in a manner incongruous with genome tethering. The absence of the dimerization domain in IE19 may allow this much smaller protein to oligomerize, as has been shown to be essential for the function of the other viral tethers (3, 4, 8–13). Structural studies of IE19, perhaps including viral and cellular DNAs, could begin to define why IE19 can function as a tether to support S-phase infections and why IE1 cannot.

While the impact of S-phase infections on the clinical outcomes of HCMV infection is unknown, persistence and latency are clearly required for clinical manifestations of HCMV infection. HCMV genomes are found in the majority of glioblastoma multiforme (GBM) tumors where it persistently infects GBM tumor cells (72, 73). While not considered oncogenic, HCMV is described as oncomodulatory (74, 75). HCMV renders GBMs resistant to chemotherapy and induces a stem-cell-like phenotype (76). The virus also encodes multiple proteins with functions similar or identical to established viral oncoproteins (75, 77–79). Reactivation of lifelong latent infection causes debilitating disease (80, 81). Provocatively, upon the first demonstration of IE1 mitotic chromosome binding, the authors commented this was a function “expected for a viral protein involved in the maintenance of a putative plasmid state for HCMV viral DNA during latency” (15). Indeed, UL123 exon 4, which encodes the CTD/NBM critical for viral tethering during S-phase infections, has also been implicated in viral genome maintenance during latency (57), and we hypothesize that the only UL123 exon 4-containing isoform to likely play this role is IE19. For HPV, EBV, and KSHV, viral genome tethering is absolutely required for the persistence, latency, and oncogenesis (3, 82). Therefore, we hypothesize that IE19-mediated tethering is required for HCMV-persistent infection of GBM cells and for latency. The absolute requirement of viral genome tethering for persistence and oncogenesis of HPV, EBV, and KSHV means that treatments that inhibit this process for those viruses, as well as HCMV, could be useful chemotherapies and perhaps even help rid patients of lifelong infections.

Finally, HCMV IE19 is a novel twist on the known viral tethering proteins. IE19 is a tether from a virus that is not (to our knowledge) directly oncogenic. Its functional domains are inverted compared to E2, EBNA1, and LANA, with IE19 having the CTD in the C-terminus and the (presumptive) viral genome-binding motif near the N-terminus. Furthermore, the requirement for each of the three lysine residues in exon 2 might impart a novel way for a tether to interact with its viral genome. These differences make IE19 a unique example of a viral tether worthy of further study.

## MATERIALS AND METHODS

### Plasmids and mutagenesis

Expression constructs for N-terminally 3×FLAG tagged wild-type IE1 (pSG5-3XFLAG-IE1), IE19 (pSG5-3XFLAG-IE19), and IE19 lacking the CTD (pSG5-3XFLAG-IE19 ΔCTD) have been previously described (25). Exon deletions and point mutants used in this study were constructed by PCR-directed mutagenesis using the primers listed in Table 1 or synthesized as gBlock fragments from IDT. Lentiviral vectors were constructed by subcloning 3×FLAG-tagged IE19 derivatives into pSIN-EF2-puro as EcoRI/BamHI fragments.

**TABLE 1:** Constructs and oligonucleotides used in this study

Construct	Description	Oligonucleotides	Source
3×FLAG-IE1	Wild-type allele of IE1	5′-CTCACTATAGGGCGAATTCATGGACTACAAAGACCATGACGGTGATTATAAAGATCAT GACATCGATTACAAGGATGACGATGACAAGGAGTCCTCTGCC-3′ 5′-TTTAATAAGATCTGGATCCTTACTGGTCAGCCTTGCT-3′	(25)
3×FLAG-IE1 ΔCTD	IE1 with a deletion of amino acids 476–491	5′-TAAGGATCCAGATCTTATTAAAGCAG-3′ 5′-GATCTGGATCCTTAAGAGGCGGTGGGTTC-3′	This study
3×FLAG-IE19	Wild-type allele of IE19	5′-CTCACTATAGGGCGAATTCATGGACTACAAAGACCATGACGGTGATTATAAAGATCAT GACATCGATTACAAGGATGACGATGACAAGGAGTCCTCTGCC-3′ 5′-TTTAATAAGATCTGGATCCTTACTGGTCAGCCTTGCT-3′	(25)
3×FLAG-IE19 ΔCTD	IE19 with a deletion of amino acids 157–172	5′-CTCACTATAGGGCGAATTCATGGACTACAAAGACCATGACGGTGATTATAAAGATCAT GACATCGATTACAAGGATGACGATGACAAGGAGTCCTCTGCC-3′ 5′-TTTAATAAGATCTGGATCCTTAAGAGGCGGTGGGTTC-3′	(25)
3×FLAG-IE19 G158A	IE19 G135A	5′-accgcctctggagCTaagagcacccac-3′ 5′-GTGGGTGCTCTTAGCTCCAGAGGCGGT-3′	This study
3×FLAG-IE19 H162A	IE19 H162A	5′-GGCAAGAGCACCGCTCCTATGGTGACTAG-3′ 5′-CTAGTCACCATAGGAGCGGTGCTCTTGCC-3′	This study
3×FLAG-IE19 C73A	IE19 C73A	5′-GTGACCGAGGATGCAAACGAGAACCCC-3′ 5′-GGGGTTCTCGTTTGCATCCTCGGTCAC-3′	This study
3×FLAG-IE19 Ala-Splice	IE19 A83, E84A, L85A, E86A, S87A, P88A	5′-GAGAAAGATGTCCTGGCAGCAGCCGCAGCCGCAGTACCCGCGACTATC-3′ 5′-GATAGTCGCGGGTACTGCGGCTGCGGCTGCTGCCAGGACATCTTTCTC-3′	This study
3×FLAG-IE19 ΔE2	IE19 with a deletion of amino acids 1–24	5′-GATGACGATGACAAGCCCGAGACACCCGTG-3′ 5′-CACGGGTGTCGCTGGGCTTGTCATCGTCATC-3′	This study
3×FLAG-IE19 ΔE2^NLS^	IE19 with amino acids 1–24 replaced with SV40 NLS	5′-GATGACGATGACAAGCCAAAGAAGAAGAGAAAGGTGCCC GAGACACCCGTG-3′ 5′-CACGGGTGTCGCTGGGCACCTTTCTCTTCTTCTTTGGCTTGTCATCGTCATC-3′	This study
3×FLAG-IE19 ΔE3^30-77^	IE19 with deletion of amino acids 30–77	5′-GAGAAAGATGTCCTGGCAGAAC-3′ 5′-GCCAGGACATCTTTCTCCACGGGTGTCTCGGGC-3′	This study
3×FLAG-IE19 ΔAD	IE19 with a deletion of amino acids 102–156	5′-TCAAGTAATTGTGGCTGGAGGCAAGAGCACC-3′ 5′-GGTGCTTTGCCTCCAGCCACAACTACTGA-3′	This study
3×FLAG-IE19 KR	IE19 K6R, K8R, K21R, K31R, K42R, K64R, K79R, K131R, K159R, K169R.	Synthesized as a gBlock fragment	This study
3×FLAG-IE19 KR^NLS^	IE19 KR allele with N-terminal SV40 NLS	5′-AAGAGAAAGGTGATGGAGTCCTCTG-3′ 5′-CACCTTTCTCTTCTTCTTTGGCTTGTCATCGTC-3′	This study
3×FLAG-IE19 KR E3 + E4	IE19 K31R, K42R, K64R, K79R, K131R, K159R, K169R	Synthesized as a gBlock fragment	This study
3×FLAG-IE19 KR E2	IE19 K6R, K8R, K21R	Synthesized as a gBlock fragment	This study
3×FLAG-IE19 KR E2^NLS^	IE19 KR E3 allele with N-terminal SV40 NLS	5′-AAGAGAAAGGTGATGGAGTCCTCTG-3′ 5′-CACCTTTCTCTTCTTCTTTGGCTTGTCATCGTC-3′	This study
3×FLAG-IE19 K6R	IE19 K6R	5′-AGGAGAAAGATGGACCCTGATAATC-3′ 5′-CCATCTTTCTCCTGGCAGAG-3′	This study
3×FLAG-IE19 K8R	IE19 K8R	5′-AAGAGAAGGATGGACCCTGATAATC-3′ 5′-GTCAGGATTATCAGGGTCCATC-3′	This study
3×FLAG-IE19 K21R	IE19 K21R	5′-GCCCTTCCTCCAGGGTGCCAC-3′ 5′-CCTGGAGGAAGGGCCCTCGTC-3′	This study

### Cells and viruses

De-identified primary human fibroblasts (NHDF; Clonetics) and HEK-293T were cultured in Dulbecco’s modified Eagle’s medium (DMEM; Sigma), supplemented with 10% fetal bovine serum (FBS; Gemini), 100 U/mL penicillin, 0.1 mg/mL streptomycin, and 2 mM L-glutamine (PSG; Sigma). Towne-ΔCTD and Towne-WTSS (IE19-null) viruses have been previously described (25, 35). The identity of these mutant viruses was confirmed by PCR and sequencing of the UL123 gene.

### Antibodies

The following antibodies were obtained from commercial sources: anti-tubulin (DM 1A; Sigma), anti-FLAG (M2; Sigma), anti-histone H2B (07-371; Sigma), and anti-histone H3 (ab1791; Abcam). The monoclonal antibody against the HCMV IE2 protein (3H9) has been previously described (83). Infrared dye 680- and 800-conjugated secondary antibodies (Li-Cor) were used for Western blotting. Alexa Fluor 488- and 594-conjugated secondary antibodies (Invitrogen) were used for immunofluorescence.

### Lentiviral transductions

Lentiviral transductions were performed as previously described (84). Briefly, recombinant lentivirus was produced in 293T cells and used to transduce NHDF cells. Transduced cells were selected with 5 µg/mL puromycin (Sigma) and subsequently maintained in a medium containing 1 µg/mL puromycin.

### Nucleosome co-immunoprecipitation

293T cells were transfected with pSIN expression vectors encoding N-terminally 3×FLAG-tagged alleles or an empty vector using Lipofectamine 2000 (Life Technologies), following the manufacturer’s instructions. At 48 hours post-transfection, the cells were harvested, washed twice with cold Dulbecco’s phosphate-buffered saline (DPBS; Invitrogen), and lysed in 1× cell lysis buffer (Cell Signaling Technologies) supplemented with 1 mM phenylmethylsulfonyl fluoride (PMSF) according to the manufacturer’s protocol. The resulting lysates were mixed with 10 µg of nucleosomes purified from HEK293T cells using a nucleosome preparation kit (Active Motif; cat #53504) according to the manufacturer’s instructions. Lysates and nucleosomes were incubated at 4°C with continuous rotation for 1 hour and then incubated with 2 µg of anti-FLAG (M2) antibody overnight at 4°C with continuous rotation. The IE19-nucleosome complexes were collected with protein A/G magnetic beads (Thermo Scientific) pre-washed twice with 1× cell lysis buffer containing 1 mM PMSF. Beads were incubated with lysates for 40 minutes at room temperature with rotation, collected using a magnetic rack, and washed twice with 1× cell lysis buffer supplemented with 1 mM PMSF. Samples were resuspended in loading buffer [50 mM Tris-HCl (pH 8.0), 2% SDS, 10% glycerol, 0.0005% bromophenol blue, and 6% β-mercaptoethanol] and boiled before being separated by SDS-PAGE and subsequently analyzed by Western blotting. Signal intensities were captured using Image Studio version 5.2 software (Li-Cor). The ratios of signal intensities for H2B IP/H2B input and H3 IP/H3 input were normalized with respect to the wild-type control.

### Western blotting

Cells were lysed in 1× cell lysis buffer supplemented with 1 mM PMSF, as described above. Subsequently, the cell lysates were treated with 2× SDS protein sample buffer and boiled at 95°C for 10 minutes prior to separation by SDS-PAGE and transferred to Optitran membranes (GE Healthcare). Membranes were blocked in 5% bovine serum albumin within Tris-buffered saline with Tween 20 (TBST) and probed with appropriate primary and secondary antibodies. Where indicated, blots bathed in water were gently scraped to remove bound antibodies before re-probing. Blots were imaged and quantitated using a LiCOR Odyssey Fc Imager with Image Studio v5.2 software.

### Indirect immunofluorescence

NHDF cells were cultured, synchronized, and/or infected for indirect immunofluorescence on glass coverslips (round, 12 mm diameter). Unless otherwise specified, cells were washed twice with cold PBS and fixed with 1% paraformaldehyde for 30 minutes at room temperature. Immunofluorescence was performed as previously described (85).

### Mitotic localization

Asynchronous NHDF cells were seeded at a density of 1 × 10^4^ cells/cm^2^ and incubated for 16 hours. The cells were then washed two times with DPBS and synchronized in the S phase by the addition of DMEM (supplemented with 10% FBS and PSG) containing 2 µg/mL aphidicolin (Sigma). Twenty-four hours later, the cells were washed three times with DPBS to release them from the S-phase blockage, and fresh medium was added to normal culture volumes and cells were incubated for an additional 12 hours to allow them to reach mitosis before being fixed with cold methanol for 10 minutes at -20°C and analyzed by indirect immunofluorescence. Images were captured using a Leica Stellaris 5 confocal microscope equipped with a HyD detector with a 63× oil objective and default settings. The LAS X acquisition software was used, employing a frame sequential data acquisition scheme utilizing 405 and 594 nm laser lines for Hoechst and Alexa Fluor 594, respectively. Images were processed using NIH FIJI/ImageJ software and Photopea online image software (https://www.photopea.com/) (86, 87). The presented images are the most representative ones from three independent biological experiments.

### G0/S-phase infection assays

G0/S-phase infection assays were performed as previously described (25). Briefly, NHDFs were seeded on glass coverslips at a density of 1 × 10^4^ cells/cm^2^ and incubated for 16 hours. NHDFs were then synchronized in G0/G1 by serum starvation or in S phase by aphidicolin treatment prior to infection with HCMV at an MOI of 0.5 for 24 hours. Infected cells were fixed and stained for IE2. Images were acquired using a Nikon Ti-Eclipse inverted wide-field microscope, captured with a CoolSnap HQ camera, and recorded through Nikon NIS Elements software (version 4.00.03). A minimum of 500 nuclei were counted for each condition. Subsequent image processing was carried out using FIJI/ImageJ and Photopea online image software.

### Data presentation and analysis

Each graph displays the means and standard deviations from three independent biological replicates. Statistical significance was determined using an unpaired two-tailed Student’s *t*-test.
